# Exploring the Biological and Phytochemical Potential of Jordan’s Flora: A Review and Update of Eight Selected Genera from Mediterranean Region

**DOI:** 10.3390/molecules29051160

**Published:** 2024-03-05

**Authors:** Manal I. Alruwad, Riham Salah El Dine, Abdallah M. Gendy, Manal M. Sabry, Hala M. El Hefnawy

**Affiliations:** 1 Department of Pharmacognosy, Faculty of Pharmacy, Cairo University, Kasr El-Aini Street, Cairo 11562, Egypt; manal.ib.ruwad@std.pharma.cu.edu.eg (M.I.A.); riham.salaheldine@pharma.cu.edu.eg (R.S.E.D.); hala.elhefnawy@pharma.cu.edu.eg (H.M.E.H.); 2 Department of Pharmacology and Toxicology, Faculty of Pharmacy, October 6 University, Giza 12585, Egypt; abdallahmohammed@o6u.edu.eg

**Keywords:** flora of Jordan, Mediterranean region, selected genera, phytochemicals, pharmacological properties

## Abstract

Jordan’s flora is known for its rich diversity, with a grand sum of 2978 plant species that span 142 families and 868 genera across four different zones. Eight genera belonging to four different plant families have been recognized for their potential natural medicinal properties within the Mediterranean region. These genera include *Chrysanthemum* L., *Onopordum* Vaill. Ex. L., *Phagnalon* Cass., and *Senecio* L. from the Asteraceae family, in addition to *Clematis* L. and *Ranunculus* L. from the Ranunculaceae family, *Anchusa* L. from the Boraginaceae family, and *Eryngium* L. from the Apiaceae family. The selected genera show a wide variety of secondary metabolites with encouraging pharmacological characteristics including antioxidant, antibacterial, cytotoxic, anti-inflammatory, antidiabetic, anti-ulcer, and neuroprotective actions. Further research on these genera and their extracts will potentially result in the formulation of novel and potent natural pharmaceuticals. Overall, Jordan’s rich flora provides a valuable resource for exploring and discovering new plant-based medicines.

## 1. Introduction

Jordan’s unique location at the crossroads of Asia, Africa, and Europe, between 29°11 N and 33°22 E, has resulted in a diverse geography and four distinct geographical zones: Mediterranean, Irano-Turanian, Saharo-Arabian, and Sudanian, with around 2978 plant spp. belonging to 868 genera. Jordan has one of the greatest global biodiversity levels [1]. The country’s flora includes medicinal and herbal plants as well as fragrant and spice-like herbs and flowers [2]. This review focuses on the phytochemical and pharmacological properties of selected genera (*Chrysanthemum* L., *Onopordum* Vaill. ex L., *Phagnalon* Cass., *Senecio* L. *Clematis* L. *Ranunculus* L., *Anchusa* L., and *Eryngium* L.) found in the Mediterranean region of Jordan. The Mediterranean region is primarily located in the highlands of Jordan, and is associated with altitudes above 700 m. This region receives the greatest amount of rainfall, ranging from 300 to 600 mm, and experiences the lowest mean maximum temperature, ranging from 15 to 20 °C, with minimum annual temperatures of 5 to 10 °C. The soil composition primarily consists of Terra Rossa and yellow Mediterranean soil (Rendzina), which is well-suited for rainfed arable agriculture and horticulture. Additionally, this region has the highest tree coverage [1].

The purpose of this review is to present a thorough summary of the potential medicinal properties of specific genera found in the Mediterranean region of Jordan, utilizing credible sources obtained from electronic databases up until 2022.

One of the plant genera reviewed is *Chrysanthemum* L., which is a group of almost 300 *spp.* belonging to the Asteraceae family [3] and is widely distributed in Asia and northeastern Europe, with most *spp.* being native to East Asia [4]. *Chrysanthemum* L. has been traditionally used in medicine for its potential to improve liver function and reduce inflammation [5]. In Jordan, two *spp.* of *Chrysanthemum* L. are present, known as *Ch. segetum* L. (syn. *Glebionis segetum* (L.) Fourr. and *Ch. coronarium* L. (syn. *Glebionis coronarium* L.) [6]. Another genus in the same family, *Onopordum* Vaill. Ex. L., is utilized as food and in traditional medicine in many nations. It has antibacterial, hemostatic, and hypotensive properties and is used to treat skin cancer [7]. *Onopordum* Vaill. ex L has five identified species in Jordan, namely *O. alexandrinum* Boiss., *O. carduiforme* Boiss., *O. cynarocephalum* Boiss & Blanche., *O. heretacanthum* C.A. Mey, and *O. palaestinum* Eig. [8]. *Phagnalon* Cass. is a genus within the Asteraceae family that has long been utilized in conventional medicines to alleviate headaches, toothaches, and asthma symptoms [9]. The genus comprises several spp. with potential medicinal properties, but in Jordan, only one species of this genus, *Ph. rupestre* L., is present. However, these species have a wide distribution throughout Jordan [10]. *Senecio* L. is another genus within the *Asteraceae* family, which comprises almost 1500 spp. worldwide. It is employed in conventional medicine as an emmenagogue, anti-inflammatory, vasodilator, and hypoglycemic drug [11]. Several spp. of *Senecio* L. are found in Jordan including *S. vulgaris* L., *S. flavus* sch.Bip., *S. glaucus* L. subsp. *coronopofolius* C. Alexander, and *S. leucanthemifolius* subsp*. vernalis* Poir. [10]. Genus *Clematis* L. comprises about 300 spp., which are distributed worldwide [12]. Many of these species are known for their medicinal properties and are used as a diuretic, antidysentery, snake bite antidote, antimalarial, and in the treatment of bone illnesses, chronic skin disorders, rheumatic pain, fever, eye infections, gonorrheal symptoms, gout, and varicosity as well as to treat blisters, festering wounds, and ulcers [13]. In Jordan, two *Clematis* spp., namely *C. cirrhosa* L. and *C. flammula* L., are found [14]. Genus *Ranunculus* consists of around 600 spp. and has been utilized traditionally to treat a variety of illnesses including fevers, conjunctivitis, abscesses, and rheumatism. It also has antihemorrhagic, anti-spasmodic, and diaphoretic properties, and has been used to treat conditions like malaria, scrofula, snake and scorpion bites, and acute hepatitis [15]. In Jordan, approximately seven *Ranunculus* L. spp. have been identified, namely *R. arvensis* L., *R. asiaticus* L., *R. cornutus* DC., *R. chius* DC., *R. sceleratus* L., *R. muricatus* L., and *R. paludusus* Poir. [14]. Another genus included in this review is *Anchusa* L., which also includes 15 genera that are indigenous to temperate and subtropical regions of the Old World. Folk medicine has employed various *Anchusa species* to treat ailments such as open wounds and cuts, rheumatism, arthritis, gout, stomach diseases, and weight loss [16,17,18,19]. In Jordan, there are five common spp. of *Anchusa* L., namely *A. undulate* L., *A. strigosa* Banks & Sol., *A. azurea* Mill., *A. milleri* Lam. Ex Spreng, and *A. aegyptiaca* (L.) A.DC. [10]. Finally, genus *Eryngium* L., with approximately 250 spp. distributed worldwide, is well-known for its anti-inflammatory and diuretic characteristics as well as its ability to treat a number of diseases like hypertension, digestive issues, asthma, burns, fevers, diarrhea, and malaria [20,21,22]. Jordan has four identified *Eryngium* L. species including *E. creticum* Lam., *E. glomeratum* Lam., *E. falcatum* F. Delaroche, and *E. maritimum* L. These species are considered common and widespread in Jordan [10,14]. Table 1 provides a summary of the selected genera, and their species present in Jordan, along with their common names.

The review offers an overview of the biological and phytochemical research conducted on various plant spp., which could be a useful tool for researchers looking to further explore and comprehend the characteristics and possible uses of these plants. The review lays the groundwork for future research that can uncover novel applications for these species by highlighting the existing understanding of the chemical constituents and therapeutic characteristics of these plants. Furthermore, preclinical, and clinical studies of these plants could help to identify the most promising candidates for drug development, determine appropriate dosages and formulations, and evaluate the effectiveness and safety of these natural compounds for use in people. Such studies could also help to validate the traditional uses of these plants and provide scientific evidence for their therapeutic potential. In addition, the development of novel drugs or natural health products based on these plants could have significant economic benefits, particularly for communities that fulfil their medical requirements with conventional plant-based medication. It is also critical to keep in mind that more research could be required to properly comprehend the potential advantages and disadvantages of these plants. For instance, additional study is required to understand the pharmacokinetics and pharmacodynamics of the active chemicals found in these plants as well as their modes of action. Moreover, it is essential to ensure the safety and quality of these natural products, which may require the development of standardized protocols for cultivation, harvesting, extraction, and quality control. It is important to note that there may be some species that require further investigation. Therefore, additional studies may be necessary to fully understand the potential benefits and limitations of these plants. It is important to note that there may be some species that require further investigation. Therefore, additional studies may be necessary to fully understand the potential benefits and limitations of these plants. Overall, the review provides a valuable resource for researchers interested in investigating the properties and potential applications of these plants and underscores the importance of further research in this field to fully realize the medicinal potential of plant biodiversity.

## 2. Methods

In this review, a comprehensive literature search was conducted to gather data on the biological effects and phytochemicals of different plant species. The search was performed using keywords such as *Chrysanthemum*, *Onopordum*, *Phagnalon*, *Senecio*, *Clematis*, *Ranunculus*, *Anchusa*, and *Eryngium* as well as terms related to phytochemicals, bioactive compounds, and secondary metabolites. These terms included antioxidant, antimicrobial, anti-inflammatory, cytotoxic activity, antidiabetic, neuroprotective, anti-ulcer, and cardioprotective as well as plant extract, essential oils, and pure compounds. To gather information on the phytochemicals and biological properties of various plant species, a comprehensive literature search was conducted using the Science Direct, Google Scholar, and PubMed databases. These databases are well-known for their extensive coverage of scientific literature. To ensure the reliability and validity of the information, only papers written in English between 2000 and 2022 were included in the review. In total, a minimum of 186 relevant literature reviews were identified, which were carefully evaluated and analyzed to offer a thorough overview of what is currently known about the chemical components and medicinal properties of these plant species. Studies examining applications outside of medicinal value were disregarded. PubChem was used to verify all chemical structures, and Chem Draw Professional 17.0 was used to draw them.

## 3. Results

### 3.1. Biological Activities

#### 3.1.1. Antioxidant Activities

In recent years, the pharmacological and biological properties of natural compounds have drawn increasing attention, particularly from plant sources. For thousands of years, traditional medicine has employed plants to treat a wide range of illnesses, and modern research has confirmed many of their therapeutic properties [23]. One important area of research in natural product pharmacology is the investigation of antioxidant activities [24]. Plants abound with an abundance of antioxidants, and many plant products have been assessed for their capacity to scavenge free radicals. In vitro antioxidant assays including 2,2′-azino-bis-3-ethylbenzthiazoline-6-sulfonic acid (ABTS), cupric reducing antioxidant capacity (CUPRAC), 1,1-diphenyl-2-picrylhydrazyl (DPPH) radical, ferric reducing antioxidant power (FRAP), lipid peroxidation (LPO), oxygen radical absorption capacity (ORAC), and thiobarbituric acid (TBA) assays been employed to assess the antioxidant activity of these plant compounds [25]. The evaluation of antioxidant activity is an important aspect of the study of natural products as it provides insight into their potential therapeutic uses. Table 2 summarizes the information on the antioxidant properties of particular plant species.

- Crude extracts and essential oils:

Numerous studies on the antioxidant effects of the selected plant species have been reported. For instance, a comparative study revealed that the EtOAc flower extract of *Ch. segetum* demonstrated the strongest antioxidant activity of all the extracts examined, as determined by the CUPRAC and DPPH assays. However, the extract’s antioxidant activity was not greater than that of the standard compounds including butylated hydroxyanisole (IC_50_: 6.14 µg/mL, A050 value: 5.35), butylated hydroxyltoluene (IC_50_: 12.99 µg/mL, A050 value: 8.97), and α-tocopherol (IC_50_: 13.02 µg/mL) [26]. Research conducted on *O. alexandrinum* demonstrated that both the volatile oil and the unsaponifiable fractions of the seed and aerial parts had significant antioxidant activity comparable to Trolox with a 95.07% radical scavenging effect [27]. The extracts of *O. alexandrinum* have demonstrated noteworthy properties in protecting the liver and scavenging free radicals. Ascorbic acid, silymarin, and quercetin were employed as positive controls [28]. Studies have evaluated the antioxidant activity of various extracts of *clematis*. For instance, a recent study discovered robust antioxidant activity in the essential oil of *C. cirrhosa*. As positive controls, ascorbic acid and Trolox were utilized, as stated in the study [29]. Additionally, previous research has shown that *C. flammula* extracts possess antioxidant properties compared to vitamin E, with an IC_50_ of 190 µg/mL as assessed by the DPPH assay [30]. A comparative analysis showed that the *C. cirrhosa* methanol extract demonstrated a noteworthy overall antioxidant ability, a slightly higher reducing power, and a noteworthy ability to scavenge DPPH free radicals. However, the hydromethanol extract was observed to possess a slightly better ABTS^•+^ scavenging capacity compared to the methanol extract [31]. The antioxidant activity of *Ranunculus* species, especially *R. sceleratus*, has been extensively studied. In a study by Shahid (2013), various fractions of *R. sceleratus* were evaluated for their antioxidant capacities. The ethyl acetate soluble fraction demonstrated the maximum suppression of DPPH radicals, FRAP value, and overall antioxidant activity in relation to ascorbic acid and in comparison to other fractions, having an IC_50_ of 58.90 μg/mL [32]. However, a different study involving four *Ranunculus* spp. including *R. ficaria*, *R. sardous*, *R. bulbosus*, and *R. sceleratus* reported contrasting results. Among these species, *R. sceleratus* exhibited the lowest antioxidant activity when assessed using various in vitro techniques such as DPPH, TEAC, FRAP, CUPRAC, and SNP. The positive control Trolox demonstrated IC_50_ values of 17.4 µg/mL and 50.4 µg/mL in the TEAC and DPPH assays, respectively [33]. Nevertheless, other studies have reported potent antioxidant activity for *R. sceleratus*. Solanki et al. (2020) found that *R. sceleratus* displayed the highest H_2_O_2_ scavenging activity as well as the highest ABTS and DPPH radical scavenging activity. In all of the conducted assays, ascorbic acid was utilized as a control [34]. Additionally, Serag et al. (2020) discovered that the crude extract derived from *R. sceleratus* demonstrated an even stronger antioxidant activity compared to the commercially available antioxidant catechol, with an impressive scavenging activity of 84.35% [35]. Numerous investigations have assessed the antioxidant capacity of various *Anchusa* species. Research conducted on *A. undulata* subsp. hybrida showed that it displayed significant antioxidant activity based on four different test assays including the total antioxidant activity, phosphomolybdenum method, antiradical activity, and reducing power activity [36]. Another study using the ABTS and DPPH radical scavenging assays showed that *A. undulata* subsp. hybrida displayed natural antioxidants [37]. In a study conducted on *A. strigosa*, it was observed that the extract from this plant exhibited significant inhibition of *β*-carotene bleaching when compared to 9.5 µg/mL of rutin. Additionally, the floral extract displayed moderate activity against DPPH radicals in comparison to 1.48 µg/mL of ascorbic acid, the positive control [38]. Moreover, the root extract of *A. italica* demonstrated superior antioxidant activity in comparison to the leaf extract and ascorbic acid (with an IC_50_ of 0.121 µg/mL) [39]. Several investigations have been carried out to examine the antioxidant capacity of different species of *Eryngium* found in Jordanian flora. Among these species, *E. creticum* has shown potential in preventing disorders associated with oxidative stress, as its ability to scavenge ABTS radicals has been shown to enhance with the concentration of aqueous extract applied. The total antioxidant activity of different parts of *E. creticum* varied significantly, indicating the presence of several antioxidant and bioactive chemicals [40]. The findings from three in vitro antioxidant assays, namely DPPH, Ferrozine, and H_2_O_2_ showed notable antioxidant activity in *E. creticum*’s ethanolic and aqueous extracts [41]. Another study found that varying concentrations of ethanol in *E. creticum* extracts showed different antioxidant capabilities, with the 40% ethanol extracts exhibiting the most iron chelating activity and 80% ethanol extracts exhibiting the highest DPPH scavenging activity. As a positive control, ascorbic acid was employed [42]. Additionally, it has been found that strong antioxidant activity was demonstrated by *E. maritimum*. Both the essential oil and the oxygenated fraction of *E. maritimum* demonstrated significant antioxidant activity according to the DPPH and ABTS radical-scavenging activity tests [43]. Moreover, essential oils extracted from *E. maritimum* fruits exhibited a significantly higher radical scavenging capacity compared to the Trolox control [44]. The antioxidant activity of many extracts made from the aerial sections of *E. serbicum* and *E. maritimum* was compared, and it was discovered that the aqueous extract of *E. serbicum* had higher antioxidant activity. The IC_50_ values obtained from the DPPH assay for the positive controls were 0.093 mg/mL for butylatedhydroxyanisole and 0.054 mg/mL for ascorbic acid. Additionally, the ABTS value for the positive control butylatedhydroxyanisole was 2.66 mg AA/g [45]. Among the five eco-friendly extraction methods tested on *E. maritimum*, the supercritical fluid extraction (SFE) and 80% ethanol reflux extracts exhibited the highest efficacy in inhibiting xanthine oxidase activity. This was followed by the aqueous reflux extraction method. Furthermore, the DPPH study revealed that the aqueous extracts had the strongest antioxidant activity, with a result exceeding 70%. Quercetin was used as a positive control with a xanthine oxidase inhibition of 102%, whereas in the DPPH assay, ascorbic acid was utilized as a positive control with a 95% inhibition rate. These findings can be attributed to the metabolite composition within the extracts [46].

- Pure compounds

Muriolide (1), a naturally occurring lactone isolated from *R. muricatus*, has been demonstrated to be an effective radical scavenger in the physiological environment [47]. Another compound isolated from *R. muricatus*, muricazine (2), a novel natural hydrazine derivative, has shown significant potential in scavenging the DPPH free radical. However, it exhibited only moderate inhibitory activity against the enzymes lipoxygenase and urease [48]. Acacetin-7-*O*-galacturonide (3), a flavone glycoside identified in *O. alexandrinum*, has demonstrated significant properties as a free radical scavenger and hepatoprotective agent and was compared to positive controls such as ascorbic acid, silymarin, and quercetin [28]. 

The findings on the antioxidant properties of Jordanian flora suggest that further research is warranted to explore their potential therapeutic applications. Subsequent research endeavors may concentrate on clarifying the modes of operation of these substances, evaluating their safety and efficacy in vivo, and investigating their possible application in the management of a range of oxidative stress-related illnesses. Additionally, the development of novel extraction and purification techniques could help improve the yield and quality of these compounds, making them more viable for therapeutic applications.

#### 3.1.2. Antimicrobial Activities

Investigating various extracts obtained from traditional medicinal plants as possible sources of novel antimicrobial agents has drawn more attention in recent years [49]. Because many pathogenic microbes have developed resistance, the search for novel antimicrobial drugs is a crucial study area [50]. Several bioassays are used such as broth or agar dilution, disc-diffusion, well diffusion, flow cytofluorometric, and bioluminescent methods [51]. The selected plants mentioned in the review demonstrate antibacterial and antifungal activities against different strains of bacteria and fungi. *Ch. coronarium*, *S. vulgaris*, *S. leucanthemifolius*, *C. flammula*, *R. sceleratus*, *R. arvensis*, *A. strigosa*, *A. azurea*, *E. creticum*, *E. glomeratum*, *E. maritimum*, and *E. falcatum* exhibit antibacterial activity against Gram-positive bacteria. These plants have shown inhibitory effects against bacteria such as *Staphylococcus aureus*, *Enterococcus faecalis*, *Corynebacterium xerosis*, *Bacillus subtilis*, and *Proteus mirabilis*. Additionally, some plants such as *C. flammula* and *R. sceleratus* also demonstrate antifungal activity against certain fungi such as *Candida albicans* and dermatophytic strains. *E. glomeratum* and *E. maritimum* exhibit antibacterial activity against multiresistant *Pseudomonas aeruginosa*, which is a Gram-negative bacterium. However, the majority of the plants mentioned do not specifically target Gram-negative bacteria. Regarding fungi, *R. sceleratus*, *R. arvensis*, *E. creticum*, *E. maritimum*, and *R. muricatus* demonstrate antifungal activity against various species such as *Trichophyton mentagrophytes*, *Microsporum fulvum*, *Microsporum gypseum*, *Microsporum canis*, *Fusarium solani*, and *Aspergillus niger*. Moreover, *Ch. coronarium* and *A. azurea* exhibit antifungal activity against *Candida albicans*. These plants show varying degrees of antibacterial and antifungal activities, with a focus on Gram-positive bacteria and certain fungal strains. Only a few plants demonstrate significant antibacterial activity against Gram-negative bacteria. This review will cover studies identified from prior research investigations as possible antimicrobial agents. Table 3 summarizes the information regarding the antimicrobial activities of the selected plant species.

- Crude extracts and essential oils

Research has shown that *Ch. coronarium* essential oil can inhibit the formation of hyphal colonies on agricultural pathogens and has good antibacterial properties against Gram-positive bacteria [52,53]. As per the study carried out by Loizzo et al. in 2004, it has been found that the methanol extract obtained from *S. vulgaris* possesses antibacterial properties against Gram-positive bacteria, while its effectiveness against fungi is limited [54]. In the meantime, research has been undertaken on *S. leucanthemifolius*’s antibacterial and antifungal capabilities against seven distinct pathogenic organisms. High antibacterial activity was demonstrated by the ethyl acetate extract against *S. aureus*, while the *n*-hexane extract has demonstrated notable antifungal properties against the dermatophytes *T. tonsurans* and *M. gypseum*. In a previous in vitro study, the antibacterial and antifungal effects of alcohol extracts obtained from 38 species were evaluated, and it was found that *C. flammula* exhibited inhibitory effects against the growth of six bacterial spp.: *E. faecalis*, *P. mirabilis*, *L. monocytogenes*, *P. aeruginosa*, *C. jejuni*, and *C. xerosis* [57]. Additionally, the ethanol extract of *C. flammula* has demonstrated potential antifungal and anti-biofilm activities against *C. albicans*. The extract was found to limit the adhesion, proliferation, and elongation of germ tubes and hyphae, thus halting the formation and development of biofilm [58]. *Ranunculus* species have demonstrated significant antibacterial and antifungal effects. The essential oils of *R. sceleratus* have exhibited moderate antimicrobial activity [59]. Additionally, it was discovered that five dermatophytic strains could be effectively inhibited by *R. sceleratus* chloroform extracts [60]. In vitro studies have also examined the antifungal activity of *R. sceleratus*’s aqueous extract against *Aternarias* species, which are responsible for crucifer leaf blight, and it has been found to be a source of biofungicide against the tested fungus [61]. Furthermore, the ethanol extract of *R. sceleratus* exhibited the highest level of inhibitory activity against *A. baumannii*, *A. niger*, *B. subtilis*, *P. aeruginosa*, *S. aureus*, and *S. cerevisiae* [62]. In research carried out by Hachelaf et al. in 2013, it was discovered that the aqueous extract of *R. arvensis* demonstrated potent antifungal properties against *C. albicans* [63]. Moreover, research has demonstrated that the essential oil extracted from *R. arvensis* exhibits a notable ability to impede the growth of various bacteria including *S. aureus*, *E. coli*, *Enterobacter* sp., and *P. vulgaris* [64]. The dichloromethane fraction of *R. arvensis* has also been found to possess antibacterial activity against four microorganisms and has activity against *M. canis* and *F. solani* [65]. Research showed that *R. muricatus*’s ethyl acetate fraction exhibited the strongest cytotoxic effect against *S. aureus* and *A. niger*, while the *n*-hexane fraction demonstrated the best antifungal activity [66]. Earlier research has explored the antibacterial properties of total lipids extracted from *A. strigosa* against various bacterial strains. The findings indicate that these lipids are more potent against Gram-positive microorganisms than Gram-negative ones [67]. On the other hand, the essential oil of *A. strigosa* demonstrated strong antibacterial activity against both Gram-positive and Gram-negative bacteria at high concentrations (2 and 5 mg/mL). Furthermore, *A. strigosa*’s essential oils outperformed the fixed oils in their ability to combat both Gram-positive and Gram-negative bacteria [68]. The alcohol extract of *A. strigosa* demonstrated greater efficacy in inhibiting the growth of specific bacterial strains, namely *S. salivarius* and *S. pyogenes*, compared to the aqueous extract [69]. It has been discovered that *A. azurea* possesses dose-dependent inhibitory effects on *B. cereus β*-lactamase. According to the findings, the ethyl acetate extract had a very high inhibitory effect at a dose of 10 mg, with 68% inhibition using clavulanic acid as a positive control [70]. Additionally, extracts from *A. azurea* demonstrated in vitro inhibitory efficacy against seven bacterial strains as well as *C. albicans*, with the leaf ethanol extract showing the minimum inhibitory concentration against *E. coli* [39]. According to comparative research, *E. creticum* extracts from two harvest seasons revealed notable antibacterial activity against various bacteria. Gram-positive strains exhibited greater sensitivity. The aqueous extract displayed stronger antibacterial effects on *S. epidermidis* than the ethanol extract. MIC values were 5 mg/mL for the first period and 27.9 mg/mL for the second period [71]. Furthermore, it was discovered that extracts from *E. creticum* had a greater than 95% inhibitory effect on the growth of *B. cinerea* and *F. oxysporum* [72]. *E. glomeratum* essential oils have demonstrated strong antibacterial activity against multiresistant *P. aeruginosa* [73]. *E. maritimum*, on the other hand, has demonstrated promise as an antibacterial agent; the fruit and leaf essential oils have demonstrated notable efficacy against *S. aureus* and *T. mentagophytes*. Additionally, the essential oil extracted from the leaves showed some modest efficacy against *E. coli* and *C. albicans* [74]. Further studies have examined the antibacterial and antifungal activities of *E. maritimum* extracts against selected pathogenic bacteria and fungi, with all extracts demonstrating higher activity, particularly against *Bacillus cereus*. While the ethyl acetate extract demonstrated the strongest action against all of the tested fungus, particularly *A. flavus*, the methanol and *n*-butanol extracts were effective against *P. aeruginosa* [75]. Three species of Eryngium (*E. planum*, *E. campestre*, and *E. maritimum*) were also examined for their antibacterial activity. It was discovered that the ethanol extracts suppressed the growth of *T. mentagrophytes* dermatophyte strains, which cause fungal foot infections [76]. In one study, five distinct extraction methods were compared to evaluate their antibacterial activity using *E. maritimum* aerial parts. The results revealed that the supercritical fluid extraction (SFE) extract exhibited inhibitory effects against all strains of *P. acnes*. On the other hand, the 80% ethanol reflux extract only showed inhibition against the clinical strain N896 of *P. acnes*. [46]. Finally, it has been demonstrated that *E. falcatum* exhibits modest antibacterial activity against *S. epidermidis* and of *S. aureus* [77].

- Pure compounds

Upon reviewing the available literature, it was found that only a single study has reported on the antimicrobial properties of substances that have been identified from the studied species. One such compound, namely 2,3-dihydro-3*β*-hydroxyeuparin 3-*O*-glucopyranoside (4), was isolated from *S. glaucus* and demonstrated potent antibacterial activity against *S. aureus*, *B. subtilis*, and *E. coli*. Additionally, it exhibited antifungal activity against *C. albicans* and *C. tropicalis* [54].

Additional investigation is warranted to delve into the potential antibacterial and antifungal properties of isolated compounds derived from diverse plant species. Additionally, exploring the mechanisms of action of these compounds on bacteria and fungi as well as determining their optimal concentrations for utilization as natural antimicrobial agents would be of significant value. Moreover, it would be interesting to examine the potential synergistic effects of combining different compounds or extracts from different plant species to create more potent natural antimicrobial agents. Such studies may result in the development of new and effective therapies for bacterial and fungal illnesses.

#### 3.1.3. Cytotoxic and Antiproliferative Activities

A promising method for identifying new medications that could be utilized in conjunction with chemotherapy has been demonstrated using secondary metabolites derived from plants [78]. Today, several phytochemicals have been identified for their anti-tumor properties [79]. The cytotoxicity and antiproliferation capabilities of the plants in the chosen genera were assessed in relation to their effects on different cancer cell lines mainly using the MTT cell proliferation assay, XTT cell viability assay, sulforhodamine B assay, neutral red assay, and MTS assay. Table 4 summarizes the information on the cytotoxicity and antiproliferation activities of the selected plant species.

- Crude extracts and essential oils

Several plant species have shown potential cytotoxic properties in previous studies. The essential oil derived from *Ch. coronarium* exhibits antiproliferative characteristics and could potentially be useful in suppressing the growth of four different types of cancer cell lines (Caco-2, T47D, MCF-7, HeLa). The LD_50_ values for the essential oil ranged from 43 to 110 µg/mL. Vincristine was employed as a positive control in the study [53]. Significant antiproliferative activity was also demonstrated by *Ch. coronarium* against six human cancer cell lines: WM1361A, CACO-2, HRT18, MCF-7, T47D, and A375.S2. The range of IC_50_ values was 75.8 to 138.5 μg/mL [80]. *O. cynarocephalum* has been found to have anti-colon cancer properties, with the extract suppressing the growth of HCT-116 cells (IC_50_ 0.18 mg/mL) and HT-29 cells (IC_50_ 1.8 mg/mL) in a dose-dependent manner [81]. The acetone and chloroform extracts of O. cynarocephalum demonstrated inhibitory effects on melanoma cell lines including M14, A2058, and A375, with greater potency observed against A375 cells. The IC_50_ values for the A375 cells were 21.32 µg/mL for the acetone extract and 10.12 µg/mL for the chloroform extract [82]. The extracts of *S. vulgaris* exhibited notable and concentration-dependent cytotoxicity against Caco-2 cells. Vinblastine (2 mg/mL) was utilized as a positive control, and the methanolic and dichloromethane extracts of *S. vulgaris* displayed IC_50_ values of 34 mg/mL and 5 mg/mL, respectively [83]. In vitro, *S. leucanthemifolius* extracts inhibited various human tumor cell lines. Dichloromethane extracts inhibited large cell carcinoma (IC_50_ 20.1 μg/mL) and colorectal adenocarcinoma (IC_50_ 36.37 μg/mL), while the *n*-hexane extract showed activity against hepatocellular carcinoma. Vinblastine sulfate salt was the positive control [84]. The extract of *C. flammula* exhibited potent cytotoxicity against two human hepatoma cell lines, CHL and PLC, with IC_50_ values of 58.5 and 47.3 µg/mL, respectively [85]. The* E. creticum* extract was found to inhibit the growth of MCF7 growth by 68% to 72%. The different extracts from the four parts of *E. creticum* reduced the viability of the HeLa cell line [86,87]. The *E. glomeratum* extract has shown cytotoxicity against J774 cell lines, with a positive control of camptothecin having an IC_50_ value of 0.011 μg/mL [88], while *E. maritimum* exerted cytotoxic activity against the HepG2 and Hep2 cell lines [89].

- Pure compounds

According to a previous study, campesterol (5), isolated from *Ch. Coronarium*, was found to exhibit antiangiogenic potential [90]. A benzofuran glucoside, 2,3-dihydro-3*β*-hydroxyeuparin 3-*O*-glucopyranoside (4), isolated from *S. glaucus*, has demonstrated potent cytotoxicity against PANC-1 cancer cell lines (IC_50_ 7.5 μM) [55]. In addition, Jacaranone (6), a major active component of the dichloromethane extract obtained from *S. leucanthemifolius*, has shown remarkable activity against the COR-L23, Caco-2, C32, and HepG-2 cell lines with IC_50_ values between 2.86 and 3.85 μg/mL [84]. 

The research suggests that certain plant species and their extracts have demonstrated substantial promise as potential anticancer agents. However, additional investigation is needed to gain a complete understanding of the mechanisms of action behind these extracts and to isolate pure compounds as well as determine the optimal concentrations for use in cancer treatment. Future studies should focus on exploring the synergistic effects of combining different plant extracts or compounds to create more potent anticancer agents. Additionally, in vivo tests ought to be conducted after in vitro investigations to assess the safety as well as efficacy of these possible therapies. 

#### 3.1.4. Anti-Inflammatory Effect

Inflammation is a significant issue for human health [91], and while there are several anti-inflammatory drugs available, they may not be effective in all cases and can cause side effects such as with opioids and NSAIDs [92]. Therefore, there is a need for new plant-derived drug molecules that can help overcome these challenges. Plants have a range of phytoconstituents that possess anti-inflammatory properties and are associated with fewer side effects [93]. We present a discussion of the literature related to the selected plants and their anti-inflammatory effects. Table 5 summarizes the information on the anti-inflammatory effect of the selected plant species.

- Crude extracts and essential oils

According to Servi (2021), *Ch. coronarium* and *Ch. segetum* essential oils extracted from the aerial parts have demonstrated noteworthy anti-inflammatory properties through the inhibition of 5-lipoxygenase enzyme activity [94]. Similarly, in both in vitro and in vivo models of inflammation brought on by endotoxin-induced pro-inflammatory indicators (ET), *O. cynarocephalum* demonstrated strong anti-inflammatory properties [95]. In four mouse ulcer models, pharmacological analysis of the ethanol extract of *C. flammula* revealed a dose-dependent gastro-protective capability associated with a significant reduction in proton pump and myeloperoxidase activity [96]. Extracts from *R. sceleratus* can decrease the buildup of nitrites and may be helpful in the treatment of inflammatory illnesses brought on by high NO generation [97]. In all studies, *R. muricatus* extract’s anti-inflammatory and analgesic effectiveness in albino mice was comparable to that of the standard drug ibuprofen [98]. Alallan et al. (2018) found that extracts from *A. strigosa* have the potential to be used in the treatment of inflammatory disorders and rheumatoid arthritis [99]. The methanol extract of *A. azurea* and its *n*-butanol fraction exhibited considerable anti-inflammatory activity in a dose-dependent manner [100]. The E. maritimum extract exhibited anti-inflammatory effects by reducing circulating phagocyte proliferation and activation as well as nitrogen oxide (NO) synthesis [101]. The methanol extract of *E. maritimum* leaves showed anti-inflammatory properties and acetylcholinesterase inhibitory activity [99,102]. The results of studies evaluating the in vivo anti-inflammatory properties of eight different *Eryngium* species revealed that *E. maritimum* extracts have the most promising properties without exhibiting any obvious stomach injury. Significant activity was observed against TPA-induced ear edema [22].

- Pure compounds

Crude extracts and essential oils from various plant species possess significant anti-inflammatory properties. Some species like *Ch. coronarium* and *R. muricatus* have shown potential as sources of anti-inflammatory agents with fewer side effects. However, most studies have focused on crude extracts and only one study investigated the anti-inflammatory effects of a pure compound. Rosmarinic acid (7), obtained from *A. azurea*, demonstrated anti-inflammatory effects comparable to indomethacin in a carrageenan-induced acute inflammation model [100].

Further research must be conducted to ascertain the efficacy of individual compounds as anti-inflammatory agents.

#### 3.1.5. Antidiabetic Effects

Elevated glucose levels in the blood due to insulin secretion defects characterize diabetes mellitus, a metabolic disorder [103]. Studies have demonstrated inhibiting the metabolism of carbohydrates by enzymes such as α-glucosidase and α-amylase is a viable approach to treating diabetes [104,105]. Many studies have shown that acarbose, a medication used to treat diabetes, inhibits *α*-glucosidase and *α*-amylase, and the enzyme inhibitory activities of other substances are often compared to acarbose equivalents to evaluate their potential as antidiabetic agents [106]. Table 6 summarizes the information on the antidiabetic effect of the selected plant species.

- Crude extracts and essential oils

*S. leucanthemifolius* extracts have potential hypoglycemic activity. The *n*-butanol extract inhibited α-amylase with a value of 89.2% [56]. *C. cirrhosa* extracts have shown inhibitory activity against *α*-glucosidase and α-amylase, which are crucial in lowering postprandial glucose levels [31]. The *A. undulata* subsp. hybrid has been found to have an antidiabetic effect. Its methanol extract showed higher *α*-glucosidase inhibitor activity compared to *α*-amylase inhibition [36]. The methanol extract of *A. undulata* exhibited effectiveness as an *α*-amylase and *α*-glucosidase inhibitor, which could potentially aid in reducing postprandial hyperglycemia [37]. The *A. strigosa* extract reduced blood sugar levels significantly in a dose-dependent manner. Additionally, the serum insulin levels increased [107]. Moreover, *E. creticum* has insulin secretagogue and glucose absorption-restricting properties, which can enhance glucose homeostasis. Additionally, *E. creticum* exerts *β*-cell mass expansion bioactivity, indicating further restoration of pancreatic dysfunction [108]. 

Most of the studies conducted on the efficacy of plant extracts as antidiabetic were conducted in vitro, except for the study by Muhammed and Arı (2012) [107], which was performed on streptozotocin diabetic rats. More investigation is required to evaluate the therapeutic value of these extracts in vivo using animal models. Additionally, more studies are required to assess the toxicity of potent extracts as well as identify and characterize the active principles present in these plants for the development of new antidiabetic agents sourced from herbal resources.

#### 3.1.6. Antiulcer Agents

Peptic ulcers lead to episodic pain, discomfort, and mental distress [109]. Pharmaceutical treatments aim to reduce aggressive variables or enhance mucosal defense. Herbal therapy is increasingly accepted as a low-cost, effective, and accessible alternative to synthetic medications, with minimal side effects [110]. Herbal medicines possess gastroprotective qualities and have been employed for many years to address digestive issues and related ailments [104]. The antiulcer activity of compounds or extracts can be evaluated by employing both in vivo and in vitro models. In vivo models involve the use of animals to induce ulcers through various methods such as stress, pylorus ligation, histamine or ethanol administration and measurement of the ulcer index [111]. In vitro models involve the use of artificial gastric acid or Fordtran’s model to determine the neutralizing capacity of the prepared preparation and the measurement of gastric lesions [112]. Table 7 summarizes the information regarding the antiulcer effect of the selected plant species.

- Crude extracts and essential oils

*A. strigosa* extracts effectively minimized the ulcer index and shielded the stomach from the ulcerative agent, the ulcer index, and provided protection for the stomach from the ulcerative agent. The petroleum ether-soluble fraction exhibited the highest effectiveness, providing 91% protection and effectively lowering the ulcer index [113]. While the gastroprotective action of the *A. strigosa* extract is not yet fully understood, additional investigation is necessary to understand the in vivo mechanism. Additionally, studies are needed to evaluate the in vivo toxicity of the *A. strigosa* extract.

#### 3.1.7. Neuroprotective Effect

The term “neuroprotection” refers to methods and associated mechanisms that protect the central nervous system against neuronal damage brought on by either acute (such as a stroke) or chronic neurodegenerative illnesses [114]. The most widespread variety of neurodegenerative illnesses is Alzheimer’s disease (AD) [115]. A continuous impairment in cholinergic neurotransmission is a hallmark of AD. Alzheimer’s disease (AD) is a neurological condition characterized by degeneration of the brain, leading to symptoms such as cognitive impairment and amnesia [116]. Agents that inhibit the two main types of cholinesterase (AChE and BChE), which restore the level of acetylcholine, can be used to treat AD symptoms. As a result, cholinesterase inhibitors are crucial for the treatment of AD. Table 8 summarizes the information on the neuroprotective effect of the selected plant species.

- Crude extracts and essential oils

The *A. undulata* extract was discovered to have both acetylcholinesterase (AChE) and butyrylcholinesterase (BChE) inhibitory action in the research conducted by Sarikurkcu et al. [36]. Moreover, the methanol extract of *A. undulata* L. subsp. *hybrida* demonstrated a concentration-dependent inhibition of both AChE and BChE [37]. Both extracts of *C. cirrhosa* demonstrated significant inhibition against AChE, but the hydromethanol extract exhibited higher inhibitory activity compared to the methanol extract [31]. 

*A. undulata* and *C. cirrhosa* extracts have demonstrated significant cholinesterase inhibitory activity, indicating their potential as herbal resources for the discovery of novel anticholinesterase agents aimed at combating Alzheimer’s disease. However, further research is necessary to evaluate their in vivo potential and identify and characterize the active compounds found in this flora. 

#### 3.1.8. Miscellaneous Bioactivities

The R. muricatus extract exhibited cardiotonic activity in the isolated perfused rabbit heart [117]. The *A. italica* extract has a potent protective effect against chronic myocardial infarction injury, and the mechanisms may involve suppression of proinflammatory cytokines and the PI3K/Akt/mTOR signaling pathway [118]. Intraperitoneal injection of the *A. italica* extract was found to be an effective treatment for memory loss caused by ischemia/reperfusion as well as for related brain and serum biochemical abnormalities. This was observed over a 14-day period. The extract’s high concentration of antioxidants scavenged free radicals produced during the ischemia/reperfusion process [119]. Table 9 summarizes the information on the miscellaneous effects of the selected plant species.

### 3.2. Phytochemical Constituents

Secondary metabolites, or phytochemicals, are produced by higher plants and serve important functions such as providing defense against herbivores, stress resistance, and attracting pollinators. These compounds also have significant bioactivities for humans. Therefore, isolating and identifying phytoconstituents is a crucial step in the quest for potent natural medicines [120]. Various analytical platforms such as chromatography and spectroscopy methods including GC-MS, LC-MS, HPLC, NMR, ESI, FTIR, and UV are used to explore and characterize the chemical structures and profiles of these compounds [121]. 

#### 3.2.1. Terpenoids

Terpenoids are a diverse class of organic compounds that are categorized based on their carbon atom count into monoterpenes, sesquiterpenes, diterpenes, sesterpenes, and triterpenes. These compounds possess a wide range of structural variations and have demonstrated various biological activities. Terpenoids are widely used worldwide to treat different illnesses due to their therapeutic potential [122]. Sesquiterpene lactones, which are commonly found in plants belonging to the Asteraceae family [123], and triterpenes as well as volatile oil components were the majority of terpenoids isolated from various selected species.

**Figure 1:**
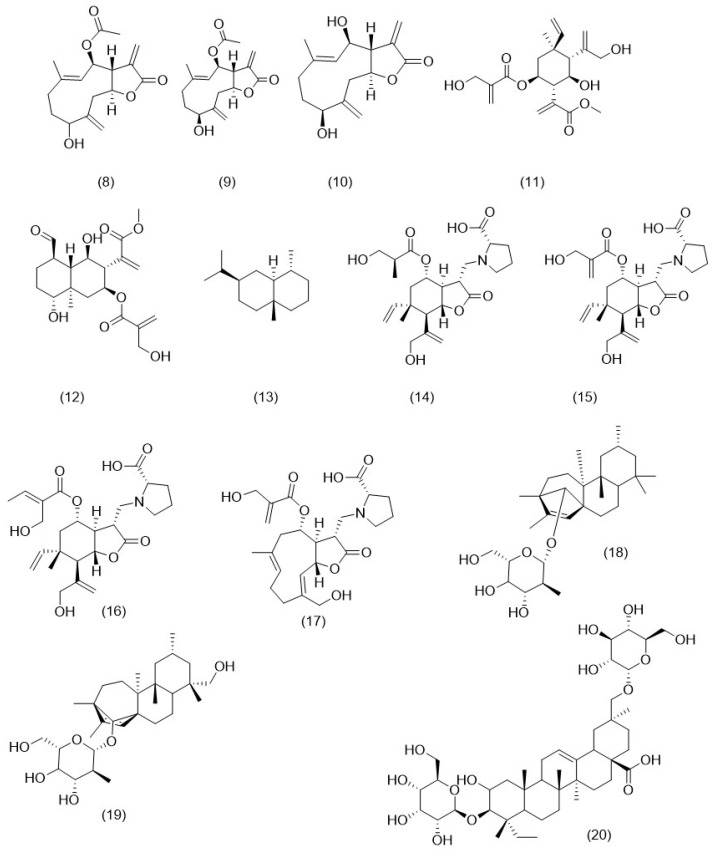

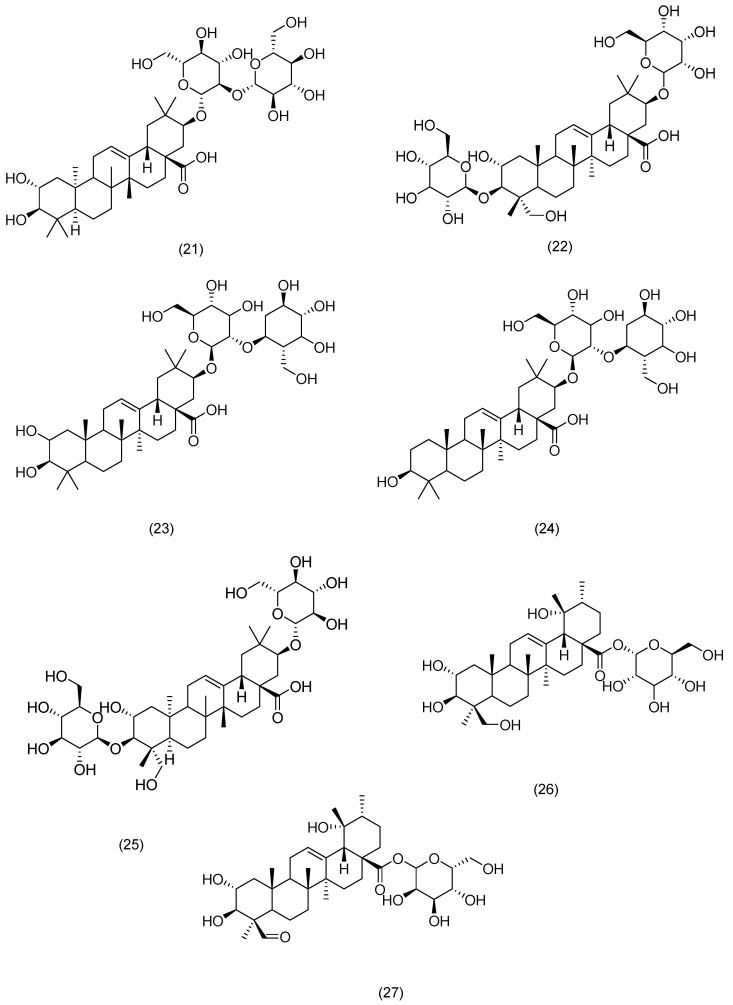

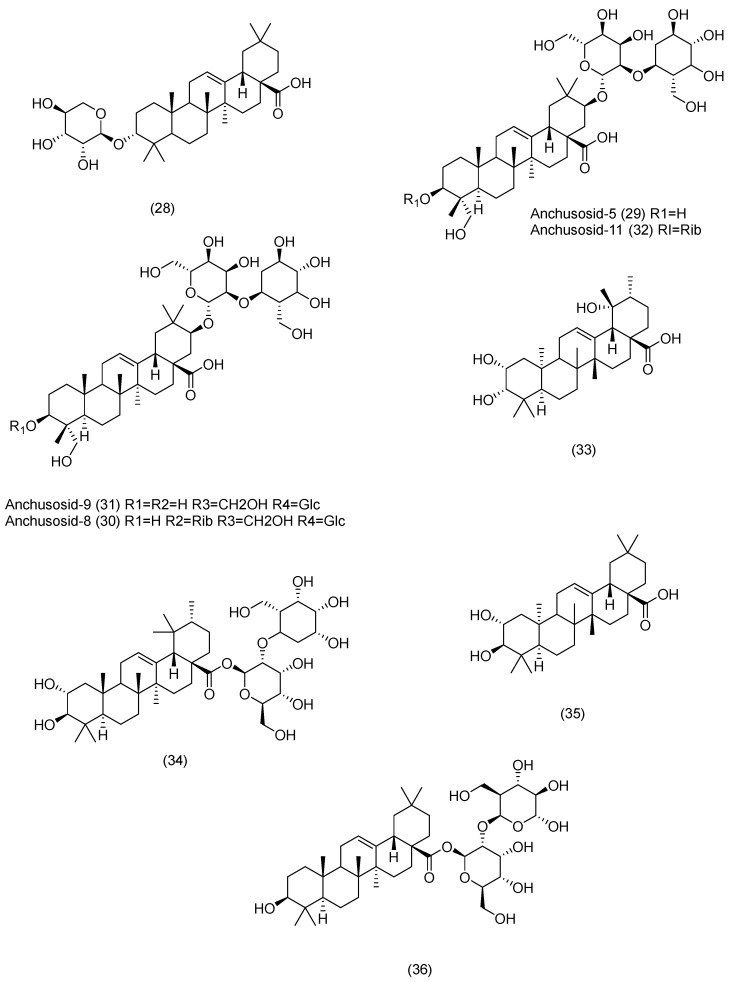

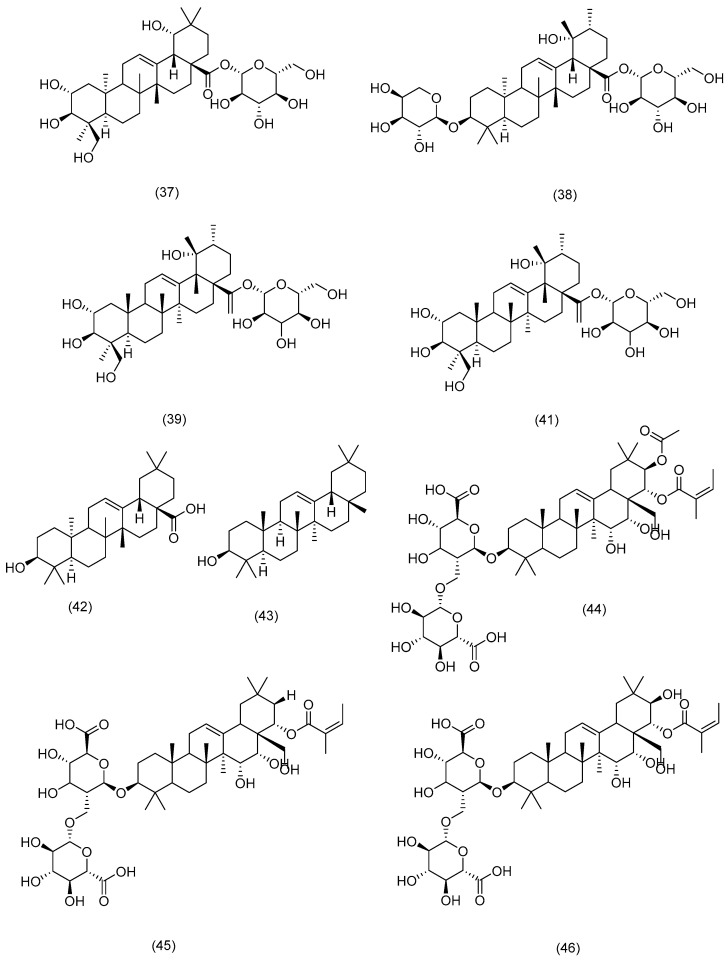
The chemical structure of the terpenoids present in the selected genera.

Three sesquiterpene lactones, namely 1-epi-dihydrochrysanolide (**8**), dihydrochrysanolide (**9**), and 1-hydroxy-1-desoxotamirin (**10**) were isolated from *Ch. coronarium* [124]. *O. cynarocephalum* was found to contain several sesquiterpenes including elemacarmanin (**11**), carmanin (**12**), and eudesmane (**13**) [82]. Meanwhile, *O. alexandrinum* was found to contain four sesquiterpene–amino acid conjugates known as onopornoids A–D comp (**14–17**) [125]. Ranunculosides A (**18**) and B (**19**), two ent-kaurane diterpene glycosides, were isolated from the aerial parts of *R. muricatus* [125]. Various triterpene glycosides have been isolated from various Anchusa species. Undulatoside, a novel triterpene glycoside identified as 3-*O*-(*β*-d-glucopyranosyl)-29-*O*-(*β*-d-glucopyranosyl)-2*α*,23-dihydroxyolean-12-en-28-oic acid (**20**), was discovered to be present in *A. undulata subsp. hybrida* [126]. *A. azurea* aerial parts yielded four triterpene glycosides: oleanazuroside 1 (**20**), oleanazuroside 2 (**21**), ursolazuroside 1 (**22**), and ursolazuroside 2 (**23**) [127]. A previous study reported a new oleanolic-type triterpene glycoside, 3*β*,21*β*-21-[(*β*-d-glucopyranosyl-(1→2)-*β*-d-glucopyranosyl)oxy]-3-hydroxyolean-12-en-28-oic acid (24) as well as five analogs: oleanazuroside 1 (**25**), oleanazuroside 2 (**21**), 24-hydroxytormentic acid ester glucoside (26), 24-epi-pinfaensin (**27**), and oleanolic acid 3-*O*-*α*-l-arabinoside (**28**) from the extract of *A. italica* whole plant [128]. Other triterpenoids isolated from *A. italica* aerial parts including oleanazuroside 2 (**21**), anchusosid-5 (**29**), anchusosid-8 (**30**), anchusosid-9 (**30**), anchusosid-11 (**32**), ursolazuroside 1 (**22**) and 2 (**23**), euscaphic acid (**33**), officinoterpenoside B (**34**), maslinic acid (**35**), sweriyunnanoside A (**36**), sericoside (**37**), ziyu-glycoside (**38**), 24-epi-pinfaensin (**27**), and 24-epi-nigaichigoside F1 (**39**) [129]. Two separate studies investigating the chemical composition of *A. strigosa* root resulted in the discovery of several triterpenes. The first identified euscaphic acid (33), euscaphic acid 28-*O*-beta-d-glucopyranoside (**40**), and oleanane glycoside 2*α*,3*β*,23,29-tetrahy-droxyolean-12-en-28-oic acid 29-*O*-*β*-d-glucopyranoside (**41**) [130] while the second identified oleanolic acid (**42**), *β*-amyrin (**43**), and crataegolic acid (**35**) [113]. Triterpene saponins identified in *E. maritimum* were 3-*O*-*β*-d-glucopyranosyl-(1→2)-*β*-d-glucuronopyranosyl-21-*O*-acetyl-22-*O*-angeloyl-R1-barrigenol (**44**), 3-*O*-*β*-d-glucopyranosyl-(1→2)-*β*-d-glucuronopyranosyl-22-*O*-angeloyl-A1-barrigenol (**45**), and 3-*O*-*β*-d-glucopyranosyl-(1→2)-*β*-d-glucuronopyranosyl-22-*O*-angeloyl-R1-barrigenol (**46**) [131]. Figure 1 illustrates the chemical structure of the terpenoids identified in the selected genera.

Essential oils (EO) are fragrant and volatile fluids derived from plant matter using the process of steam distillation [132]. These oils primarily consist of terpenes. Due to their diverse biological characteristics such as antioxidant, antibacterial, and anti-inflammatory effects, essential oils have gained significant attention in the food, cosmetic, and healthcare industries [133].

**Figure 2:**
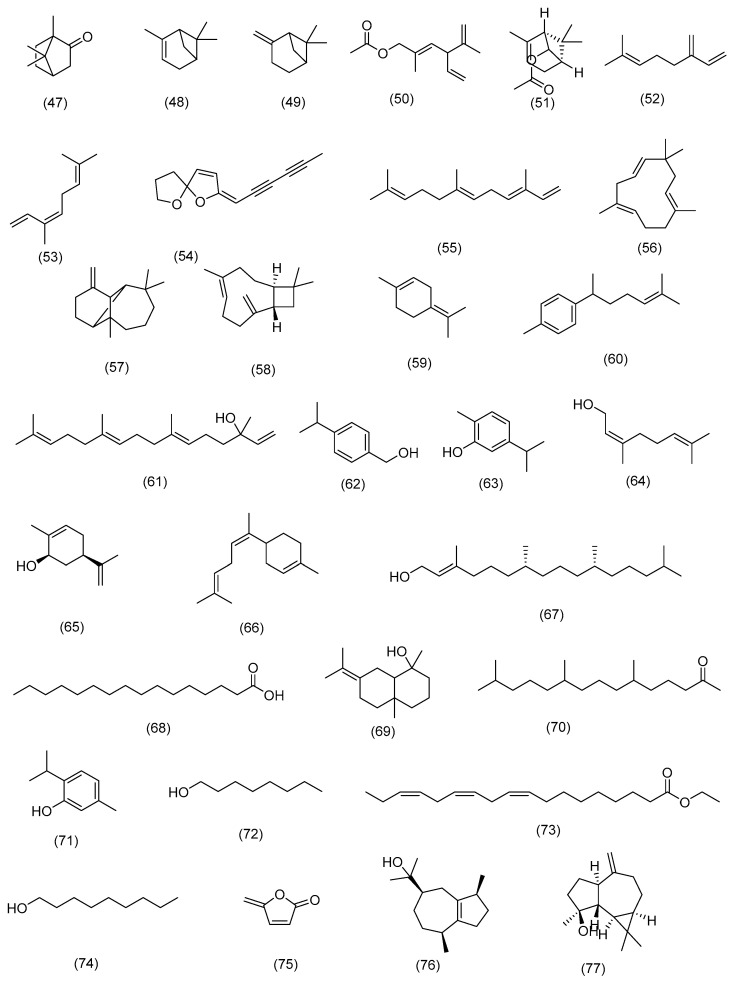

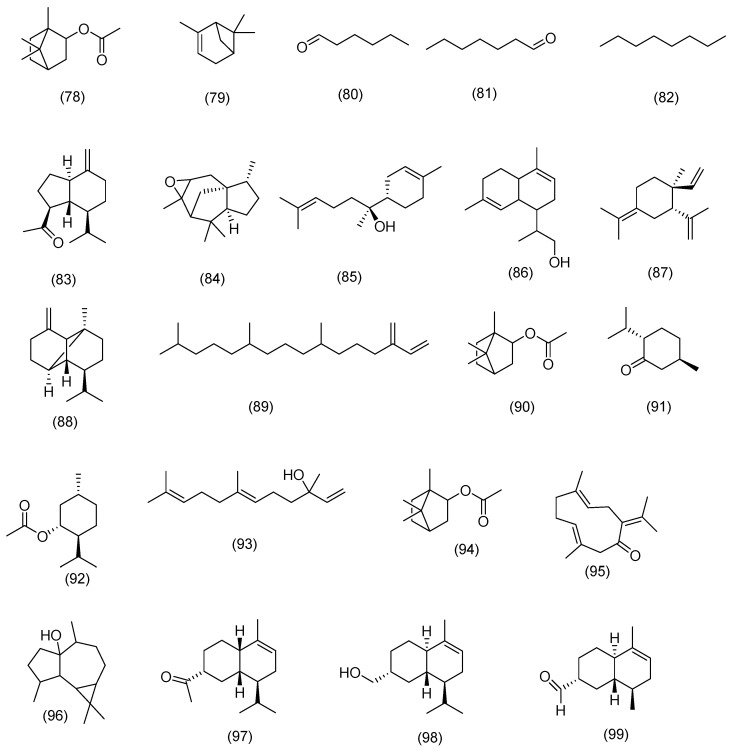
The chemical structure of the essential oil constituents present in the selected genera.

Numerous studies have investigated the chemical constitution of the essential oils (EOs) obtained from various parts of the selected species. Four investigations shed light on the chemical constitution and possible uses of essential oils derived from various *chrysanthemum*. In a study conducted by Alvarez-Castellanos et al. (2001) [52], the flowerhead oil of *Ch. coronarium* was evaluated, and its primary components were identified. These included a bicyclic monoterpene ketone camphor (**47**) as well as the bicyclic monoterpenes *α*-pinene (48) and *β*-pinene (49), and the carboxylic acid ester a lyratyl acetate (**50**) [52]. The study by Flamini et al. (2003) [134] performed headspace analyses on different parts of *Ch. coronarium* and observed differences in the pattern of volatiles emitted by each part. The study found that a bicyclic monoterpene ketone camphor (**47**) and the monoterpene *cis*-chrysanthenyl acetate (**51**) were emitted mainly by ligulate and tubular florets, while the production of the monoterpenes myrcene (**52**) and (*Z*)-ocimene (**53**) more pronounced in the flower buds. The primary ingredient of the leaves’ volatile profile was the monoterpene (Z)-ocimene (**53**), while the volatile composition of the pollen was entirely different [134]. Moreover, Senatore et al. (2004) [135] analyzed the essential oils of *Ch. coronarium* growing wild in southern Italy and identified the cyclic spiro compound *trans*-tonghaosu (**54**) with the monoterpene *cis*-chrysanthenyl acetate (**51**), the carboxylic acid ester lyratyl acetate (**50**), and a bicyclic monoterpene ketone camphor (**47**) as the main components [135]. In a separate study conducted by Marongiu et al. (2009), they isolated essential oil from *Ch. segetum* and identified sesquiterpene (*E*,*E*)-*α*-farnesene (**55**), monocyclic sesquiterpene *α*-humulene (56), and cyclic olefin *β*-longipinene (**57**) as the major components [136]. The chemical constituents of essential oils isolated from *S. vulgaris* aerial parts were examined and was found to contain **54** components in total. Among these, the most prominent compounds were monocyclic sesquiterpene *α*-humulene (**56**), polycyclic sesquiterpene (*E*)-*β*-caryophyllene (**58**), cyclohexane monoterpene terpinolene (**59**), sesquiterpene ar-curcumene (**60**), and acyclic monoterpene geranyl linalool (**61**) [137]. *S. leucanthemifolius* oil is mainly composed of monoterpenes such as *α*-hydroxy-*p*-cymen (**62**), carvacrol (**63**), acyclic monoterpene nerol (**64**), monoterpenoid carveol (**65**), and sesquiterpene* cis*-*α*-bisabolene (**66**). Notably, the *S. leucanthemifolius* oil contained higher amounts of carvacrol (**63**) and *cis*-*α*-bisabolene (**66**) compared to geranyl linalool (**61**), which is absent in the composition [11]. Two studies investigated the essential oils of different *Clematis* spp. In the first study, the essential oil of *C. cirrhosa* was examined, and a total of 12 components were isolated. The most abundant compounds in the essential oil were acyclic diterpene alcohol phytol (**67**), fatty acid palmitic acid (**68**), and terpenoid juniper camphor (**69**). Additionally, other components identified included terpenes hexahydrofarnesyl acetone (**70**) and thymol (**71**), fatty alcohols octanol (**72**) and nonanol (**74**), and the fatty acid ester linoleic acid methyl ester (**73**) [29]. In another study investigating the essential oil of *C. flammula*, the major compound identified was furan protoanemonin (**75**) [138]. According to the study by Boroomand et al. (2018), the main constituents of *R. arvensis* essential oil include polycyclic sesquiterpene guaiol (**76**), caryophyllene (**59**), terpenoid spathulenol (**77**), and the bicyclic monoterpene ketone camphor (**47**) [139]. The literature review indicates that there is considerable variation in the essential oil composition among different species of *Eryngium* as well as within the same species. This variation depends on the specific plant part from which the oil is extracted. However, some common components such as sesquiterpenes were found in the essential oils of all species. The two studies conducted on the essential oil of *E. creticum* demonstrated that the chemical composition of the oil can vary based on geographic location and extraction method. In the first study, it was found that there are seventeen components in *E. creticum* essential oil, with the bicyclic monoterpenes bornyl acetate (**78**), camphor (**47**), *α*-pinene (**48**), and monocyclic sesquiterpene germacrene D (**79**) being the major components. The oil was identified as having significant amounts of oxygenated monoterpenes [140]. In the second study by Çelik et al. (2011), which focused on *E. creticum* growing in Turkey’s Aegean region, the essential oil was found to be predominantly composed of aldehydes and oxygenated monoterpenes. The major compounds identified in this study were hexanal (**80**), heptanal (**81**), and octane (**82**) [141]. In the case of *E. glomeratum*, the essential oil extracted from the roots is primarily composed of oxygenated sesquiterpenes, while the essential oil from the aerial parts consists of oxygenated sesquiterpenes, oxygenated monoterpenes, and sesquiterpene hydrocarbons. Sesquiterpenes, particularly the monocyclic sesquiterpene germacrene D (**80**), are the main components in both oils. The root oil of *E. glomeratum* is mainly characterized by oxygenated sesquiterpenes, with *β*-oplopenone (**83**) and di-epi-cedrenoxide (**84**) as major constituents. On the other hand, the oil of the aerial parts is rich in both oxygenated sesquiterpenes and monoterpenes, with *cis*-chrysanthenyl acetate (**51**) and *α*-bisabolol (**85**) as the major components [142]. In another study by Landoulsi et al. in 2020, the volatile oil obtained from petroleum ether extracts of *E. glomeratum* exhibited high levels of oxygenated sesquiterpenes. The predominant compounds in this oil were *α*-bisabolol (**85**), 14-hydroxy-*α*-muurolene (**86**), and chrysanthenyl acetate (**52**) [88]. The chemical composition of *E. maritimum*’s essential oil is diverse, with over fifty distinct compounds identified. The chemical composition of the essential oil varies significantly between different parts of the plant. Fruit and leaf oils are primarily composed of sesquiterpenes, with germacrene D (**79**), a monocyclic sesquiterpene, being the most prevalent component in this class. Other important compounds in the fruit oil include cyclic hydrocarbon *γ*-elemene (**87**) and terpenoid *β*-ylangene (**88**), while terpenoid spathulenol (**77**) and hydrocarbon neophytadiene (**89**) are the primary components of the leaf oil. The root oil contains mainly oxygenated monoterpenes such as menthol (**90**), menthone (**91**), and the terpenoid menthyl acetate (**92**). The shoot oil has a unique composition, with pronounced amounts of some sesquiterpenes such as hydrocarbons with an eremophilane and selinane skeleton, (*E*)-nerolidol (**93**), two ketones, *β*-elemenone (**94**) and germacrone (**95**), and palustrol (**96**) being distinctive volatile constituents of *E*. *maritimum* [74]. Additionally, several sesquiterpenes including 4*β*H-muurol-9-en-15-al (**97**), 4*β*H-cadin-9-en-15-ol (**98**), and 4*β*H-cadin-9-en-15-al (**99**) have been isolated from the essential oil of the aerial parts of *E. maritimum* [43]. Overall, *E. maritimum* essential oil is a rich source of varied chemical components, each of which has a distinct composition in different plant parts. As a result, it has promising potential for use in a variety of applications including as an antioxidant [44] and antimicrobial [88]. The essential oil composition varies within and between plant species, depending on the plant part. More research is needed to understand their pharmacological properties and potential applications. Figure 2 illustrates the chemical structure of the essential oil constituents identified in the selected genera.

In summary, the plants mentioned in the study contain various types of terpenoids including sesquiterpene lactones, sesquiterpenes, triterpene glycosides, oleanolic-type triterpene glycosides, euscaphic acid, officinoterpenoside B, maslinic acid, sweriyunnanoside A, sericoside, ziyu-glycoside, ent-kaurane diterpene glycosides as well as essential oils consisting of mixtures of terpenes and terpenoids such as monoterpenes (e.g., camphor, *α*-pinene, *β*-pinene), sesquiterpenes (e.g., germacrene D), and oxygenated terpenes (e.g., linalool).

#### 3.2.2. Phytosterols 

Phytosterols are bioactive substances naturally found in plants [143]. Over 250 phytosterols have been identified, with the most common being beta-sitosterol, campesterol, and stigmasterol [144]. 

**Figure 3:**
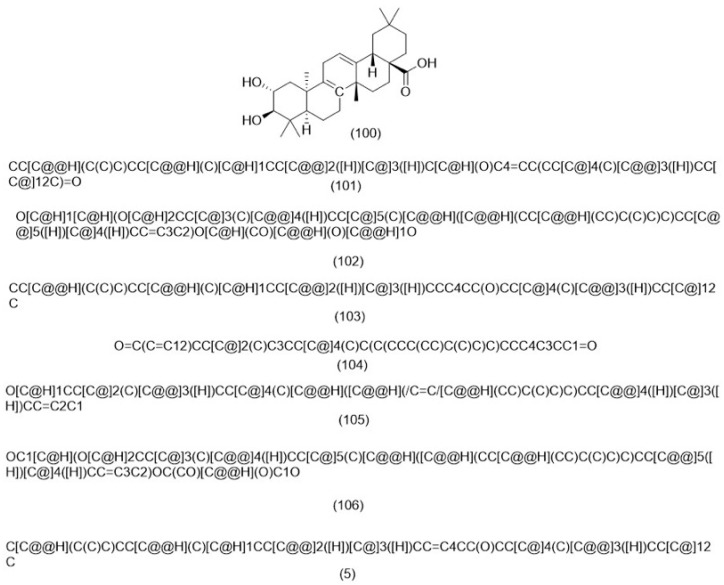
The chemical structure of the phytosterols present in the selected genera.

A variety of phytosterols were identified in five studies conducted on different plant species. However, *β*-sitosterol appears to be a common phytosterol present in several plants. Four phytosterols were isolated from *Ch. Coronarium* including stigmast-4-en-6b-o1-3-one (**100**), stigmast-4-en-6a-ol-3-one (**101**), *β*-sitosterol (**102**), and daucosterol (**103**) [145]. *β*-sitosterol (**102**) was extracted and isolated from *R. muricatus* [146]. Another investigation was carried out by Hussain et al. in 2020, who discovered the occurrence of *β*-sitosterol (**102**) and *β*-sitosterol *β*-d-glucopyranoside (**103**) in *R. muricatus* [147]. Stigmasta-4-ene-3,6-dione (**104**) and stigmasterol (**105**) were isolated from *R. sceleratus* [148]. *β*-Sitosteryl glucoside (**106**) was isolated from *A. strigosa* [113]. Furthermore, a recent study reported the isolation of *β*-sitosterol (**102**) from *E. criticum* [149]. Figure 3 illustrates the chemical structure of the phytosterols identified in the selected genera.

#### 3.2.3. Fatty Acids

Fatty acids, both free and in complex lipids, are important for energy storage and transit, membrane building, gene regulation, and mechanical, thermal, and electrical protection. PUFAs found in dietary lipids are building blocks for potent metabolites called eicosanoids [150].

**Figure 4:**
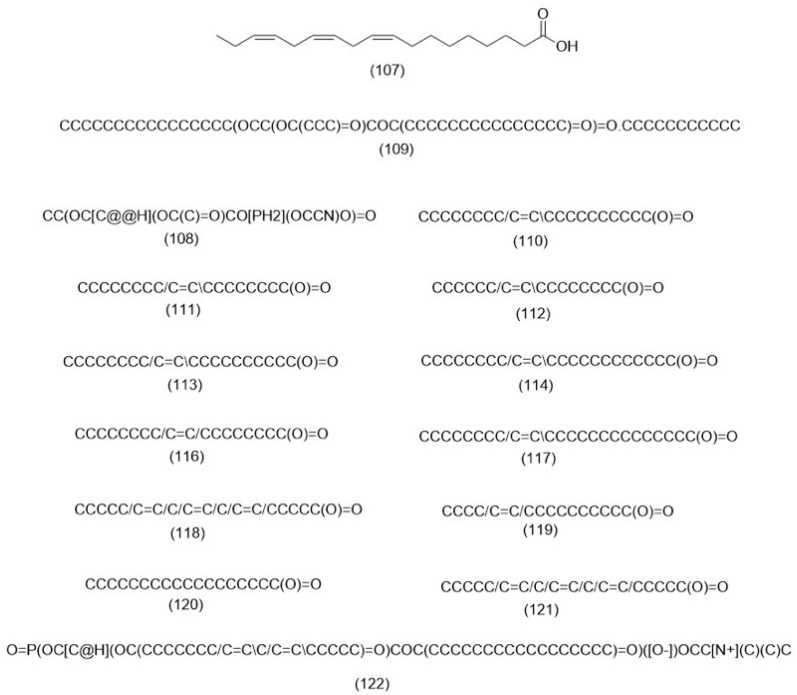
The chemical structure of the fatty acid constituents present in the selected genera.

Various studies have investigated the fatty acid content and composition of different plant species. *Ch. coronarium* was found to contain 14 different fatty acids upon analysis, with linolenic acid [107] being the primary component [151]. *A. strigosa* had a 4.42% total lipid content including two phospholipids, phosphatidyl ethanol amine (**108**) and tripalmetin (**109**), and two free fatty acids, linoleic (**110**) and palmitic acids (**68**) [67]. According to the research conducted on *A. azurea*, the plant is abundant in a variety of advantageous fatty acids. Previous research has revealed that the seeds of *A. azurea* are mainly composed of oleic (**111**), palmitic (**69**), palmitoleic (**112**), 11-eicosenoic (**113**), erucic (**114**), and two ω-9 fatty acids (**115**), with elaidic acids (**116**) being the most abundant. Additionally, minor fatty acids such as nervonic (**117**), myristic (**118**), palmitoleic (**112**), and 11-hexadecenoic acids (**119**) were identified [152]. Eleven fatty acids were extracted from *A. azurea*, and the plant was found to have high percentages of elaidic (**117**), palmitic (**69**), and linoleic acids (**110**). The other main fatty acids detected included erucic (**114**), 11-eicosenoic (**113**), stearic (**120**), and 6,9,12-octadecatrienoic acids (**121**) [153]. Research has been conducted on the fatty acid composition of *E. maritimum*, which revealed that the plant has a total oil content of 16.55%, with the most abundant fatty acids being linoleic (**110**), oleic (**111**), and palmitic acids (**68**) [154]. In a preceding investigation, it was reported that the fatty acid composition of *E. maritimum* seeds was consistent, with unsaturated fatty acids accounting for approximately 90% of the total, and with oleic (**111**), and linoleic acids (**110**) being the primary types present. Phosphatidylcholine (**122**) was found to be the primary phospholipid identified in the composition of *E. maritimum* seeds [155]. These investigations demonstrate the range of fatty acid composition and content found in different species. The identification of various fatty acid and phospholipid types can have an impact on nutrition and human health. Figure 4 illustrates the chemical structure of the fatty acids identified in the selected genera.

#### 3.2.4. Phenolic Compounds

Phenolic compounds are a heterogeneous group of secondary metabolites that feature a phenol functional group. They exhibit a wide array of significant biological impacts such as the ability to reduce inflammation, combat bacterial infections, and exhibit antioxidant activity [156]. Phenolic compounds can be categorized according to their chemical structures into several subgroups. These consist of curcuminoids, quinones, stilbenes, phenolic acids, flavonoids, tannins, coumarins, and lignans [157].

- Phenolic acids, lignans and coumarins

**Figure 5:**
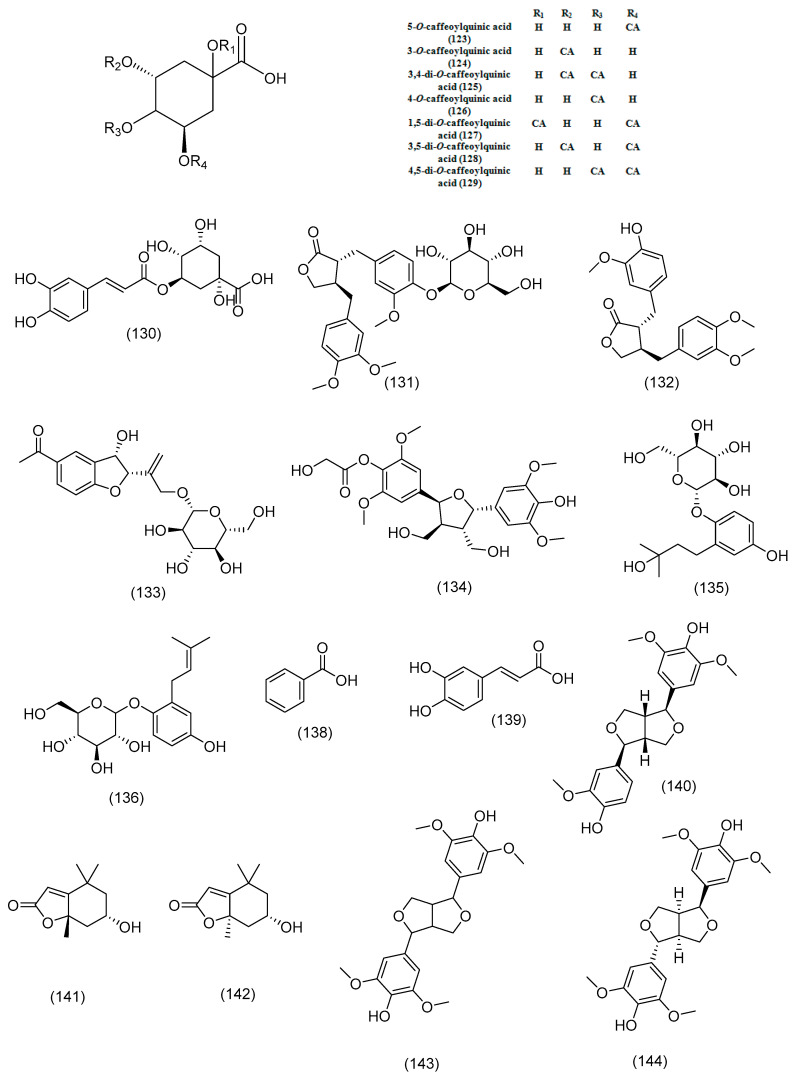

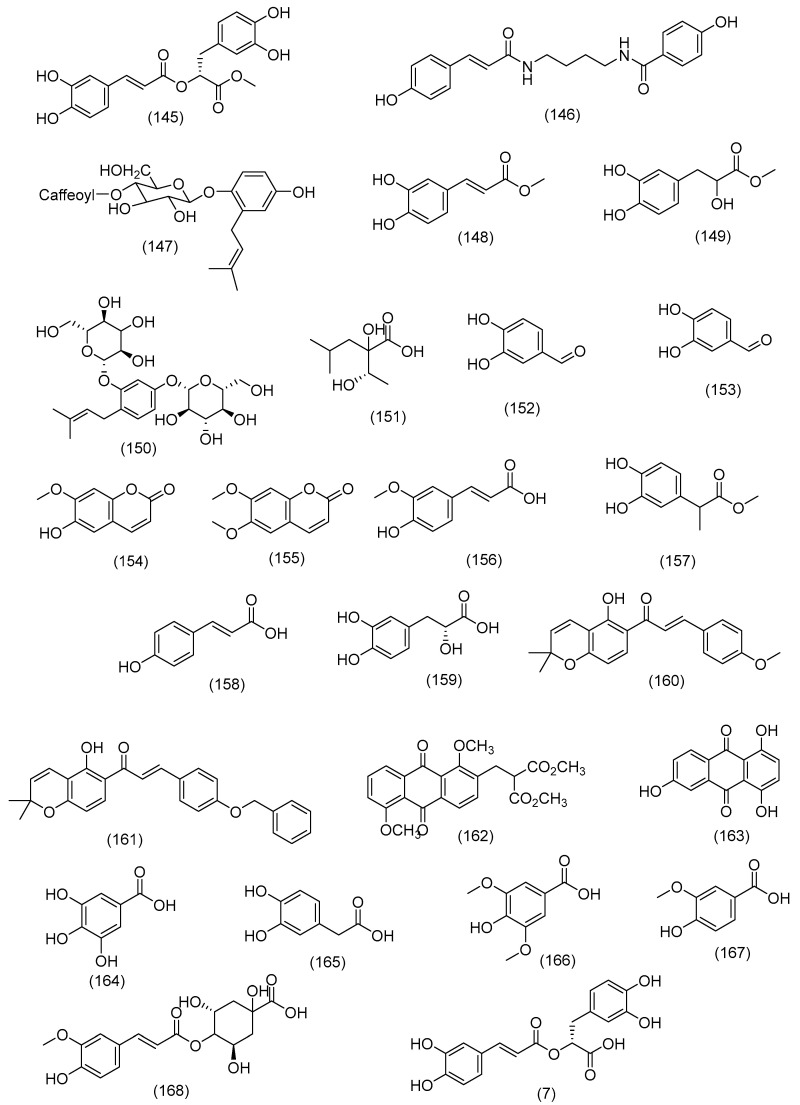
The chemical structure of phenolic acids, lignans, and coumarin constituents present in the selected genera.

Various studies have reported the presence of simple phenolics, phenolic acids (such as hydroxybenzoic acids, hydroxycinnamic acids, and coumarins), and lignans in the aforementioned species. In particular, seven caffeoylquinic acid (CQA) compounds were identified in *Ch. coronarium* and identified as 5-*O*-caffeoylquinic acid (**123**), 3-*O*-caffeoylquinic acid (**124**), 3,4-di-*O*-caffeoylquinic acid (**125**), 4-*O*-caffeoylquinic acid (126), 1,5-di-*O*-caffeoylquinic acid (**127**), 3,5-di-*O*-caffeoylquinic acid (**128**), and 4,5-di-*O*-caffeoylquinic acid (**129**) [158]. Additionally, *Ch. coronarium* leaves were found to contain significant amounts of chlorogenic acid (**130**) [159]. The lignan arctiin (**131**) was extracted from *O. alexandrinum* [28], while the two lignans, arctigenin (**132**) and arctiin (**131**), were isolated from *O. cynarocephalum* in a previous study [82]. Phenolic compounds have been identified in *Ph. rupestre* including three phenolic glycosides: 12-*O*-*β*-glucopyranosyl-9*β*,12-dihydroxytremetone (an acetophenone glycoside) (**133**), 7,7′-bis-(4-hydroxy-3,5-dimethoxyphenyl)-8,8′-dihydroxymethyl-tetrahydrofuran-4-*O*-*β*-glucopyranoside (a lignan) (**134**), and 1-*O*-*β*-glucopyranosyl-1,4-dihydroxy-2-(3′-hydroxy-3′-methylbutyl) benzene (a prenylhydroquinone glycoside) (**135**) [160]. In another study, three phenolic acid derivative compounds were also isolated from *Ph. rupestre*, namely 2-isoprenylhydroquinone-1-glucoside (**136**), 3,5-dicaffeoylquinic acid (**128**), and 3,5-dicaffeoylquinic acid methyl ester (**137**) [161]. A previous study revealed that the extracts of *C. cirrhosa* were high in benzoic acid (**138**) [31]. Several studies have reported the presence of various phenolic compounds in different species of *Anchusa* such as *A. azurea*, *A. italica*, and *A. strigosa*. In particular, *A. azurea* was found to contain chlorogenic acid (**130**), caffeic acid (**139**), and rosmarinic acid (**7**) [162,163]. Medioresinol (140), a lignan, has been detected in *A. italica* [164]. Following a phytochemical analysis of the *A. azurea* extract, a number of chemicals including epiloliolide (**141**), (–)-loliolide (**142**), (–)-dia-syringaresinol (**143**), (–)-epi-syringaresinol (**144**), methyl rosmarinate (**145**), 4-hydroxy-*N*-(4-(3-(4-hydroxyphenyl)-*E*-acryloylamino)-butyl)-benzamide (**146**), 1-*O*-*β*-d-glucopyranosyl-1,4-dihydroxy-2-(3′,3′-dimethylallyl)-benzene (**147**), methyl 3,4-dihydroxycinnamate (**148**), rosmarinic acid (**7**), and oresbiusin A (**149**) were found [152]. A study by Braca et al. (2003) reported the isolation of 7,7′-bis-(4-hydroxy-3,5-dimethoxyphenyl)-8,8′-dihydroxymethyltetrahydrofuran 4’-*O*-beta-d-glucopyranoside (**134**), a lignan, from *A. strigosa*. In addition, two phenolic compounds, 1,5-di-*O*-*β*-d-glucopyranosyloxy-2-(3′,3′-dimethylallyl) benzene (**150**) and erythro-2-hydroxy-2-(1-hydroxyethyl)-4-methyl-pentanoic acid (**151**), were also isolated from *A. strigosa* in the same study [130]. Additionally, rosmarinic acid (7) and caffeic acid (**139**) h found in the *A. strigosa* extract, according to a recent study [165]. *R. sceleratus* was found to contain protocatechuic aldehyde (**152**), protocatechuic acid (**153**), and coumarin derivatives isoscopoletin (**154**) and scoparone (**155**) [166]. In another study by Wu et al. (2013), caffeic acid (**139**), ferulic acid (**156**), methyl 3-(3′,4′-dihydroxyphenyl) lactate (**157**), *p*-coumaric acid (**158**), protocatechuic acid (**153**), and (R)-2-hydroxy-3-(3,4-dihydroxyphenyl) propionic acid (**159**) were isolated from *R. muricatus* [167]. Previous research also identified protocatechualdehyde (**152**) and the coumarin derivative isoscopoletin (**154**) in *R. muricatus* [168]. It was reported that two chalcone compounds, namely 4-methoxylonchocarpin (160) and 4-benzyloxylonchocarpin (**161**) as well as two anthraquinones, muracatanes A and B (**162**, **163**) were isolated from *R. muricatus* [147]. Several studies have identified various phenolic compounds in different species of *Eryngium*. Mejri et al. (2017) found that the most abundant compounds in the *E. maritimum* extract were caffeic acid (**139**), gallic acid (**164**), and protocatechuic acid (**153**) [169]. Furthermore, nine phenolic acids found in *E. maritimum*—chlorogenic acid (**130**), ferulic acid (**156**), 3,4-dihydroxyphenylacetic (**165**), caffeic acid (**139**), protocatechuic acid (**153**), rosmarinic (**7**), syringic (**166**), vanillic (**167**), 4-feruloylquinic acid (**168**) [170]—while (*E*)-rosmarinic acid (**7**) and an (E/Z)-rosmarinic acid mixture were isolated from *E. criticum* [149]. *E. maritimum* was found to contain the following phenolic acids: ferulic acid (**156**), caffeic acid (**139**), *p*-coumaric acid (**158**), and chlorogenic acid (**130**) [171]. Figure 5 illustrates the chemical structure of phenolic acid, lignans, and coumarin identified in the selected genera.

- Flavonoids

Flavonoids, also known as bioflavonoids, are polyphenolic compounds that are secondary metabolites in plants. They have a lean three-carbon chain and fifteen-carbon atoms, and are known for their yellow color in nature, hence their name in Latin. Flavonoids are a distinct class of plant compounds and are found in many angiosperm plant families, often serving as “flower pigments” [172]. There are different sub-classes of flavonoids such as flavans-3-ol, flavones, flavanones, flavanols, anthocyanidins, and isoflavonoids [173]. Flavonoids have been associated with health benefits when consumed through a diet rich in fruits and vegetables [174]. 

**Figure 6:**
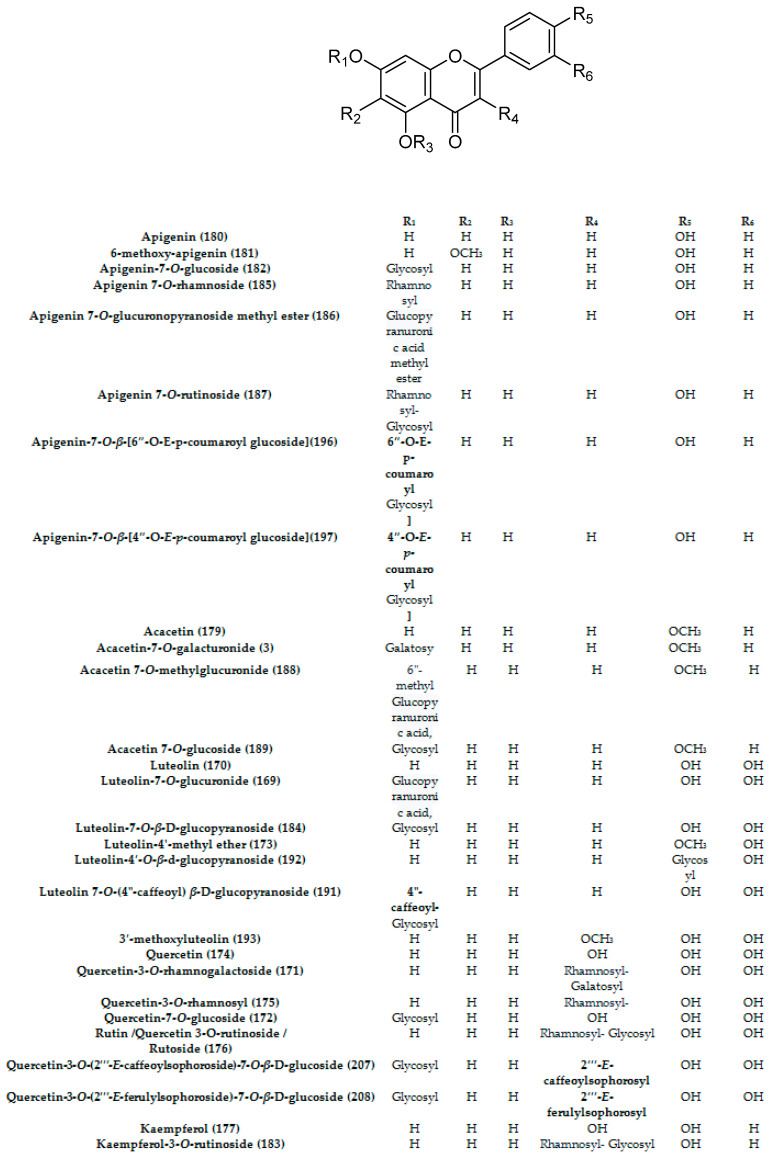

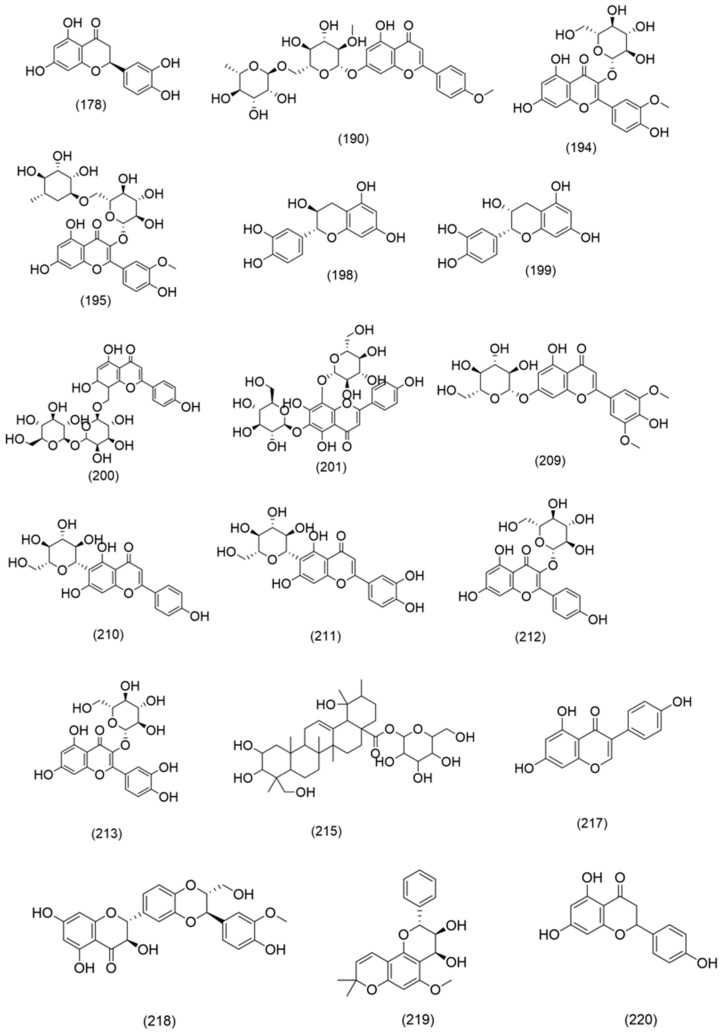
The chemical structure of flavonoids constituents present in the selected genera.

When reviewing the literature, we found that the most prevalent metabolites were flavonoids from flavones, which include apigenin and luteolin and their glucosides, and flavonols, which include quercetin and kaempferol and their glucosides. Additionally, isolated flavan-3-ols (including catechin), isoflavonoids (including genistein), and flavanones (including naringenin) were also isolated. Flavonoids luteolin-7-*O*-glucuronide (**169**), luteolin (**170**), quercetin-3-*O*-rhamnogalactoside (**171**), and quercetin-7-*O*-glucoside (**172**) were identified in the leaves of *Ch. coronarium*; luteolin-4′-methyl ether (**173**), quercetin (**174**), and quercetin-3-*O*-rhamnosyl (**175**) were identified in the flowers [151]. A second study found that the flowers of *Ch. coronarium* were rich in luteolin (**170**), whereas the leaves were high in rutin (**176**) [175]. Acacetin-7-*O*-galacturonide (3) as well as nine other known compounds including a flavonol, kaempferol (**177**); a flavonone, eriodictyol (**178**); four flavones, acacetin (**179**), apigenin (**180**), 6-methoxy-apigenin (**181**) (hispidulin), luteolin (**170**); and three glycosides, apigenin-7-*O*-glucoside (**182**), kaempferol-3-*O*-rutinoside (**183**), and luteolin-7-*O*-glucoside (**184**) were identified from *O. alexandrinum* flowers [28]. In a previous study, eight flavonoid glycosides and one acylated flavonoid glucoside were extracted from the aerial parts of *O. alexandrinum*. The identified flavonoid glycosides were apigenin 7-*O*-rhamnoside (**185**), apigenin 7-*O*-glucoside (**183**), apigenin 7-*O*-glucuronopyranoside methyl ester (**186**), apigenin 7-*O*-rutinoside (**187**), acacetin 7-*O*-methylglucuronide (**188**), acacetin 7-*O*-glucoside (**189**), linarin (**190**), and quercetin 3-*O*-rutinoside (**176**). The acylated flavonoid glucoside was luteolin 7-*O*-(4″-caffeoyl) *β*-d-glucopyranoside (**191**) [125]. A total of six flavonoids were identified in *Ph. rupestre*, which include apigenin (**181**), luteolin (**170**), apigenin 7-*O*-*β*-d-glucopyranoside (**183**), luteolin-4′-*O*-*β*-d-glucopyranoside (**192**), luteolin-7-*O*-*β*-d-glucopyranoside (**184**), and 3′-methoxyluteolin (**193**) [160]. Isorhamentin 3-*O*-*β*-d-glucoside (**194**) and isorhamentin 3-*O*-*β*-d-rutinoside (**195**) were isolated from *S. glaucus* [54]. Saidi et al.’s earlier investigation from 2019 showed that the extract of *C. flammula* contains two flavonoids, namely apigenin-7-*O*-*β*-[6″-*O*-E-*p*-coumaroyl glucoside] (**196**) and apigenin-7-*O*-*β*-[4″-*O*-E-*p*-coumaroyl glucoside] (**197**) [30]. The *C. cirrhosa* extract was found to be abundant in catechin (**198**) and epicatechin (**199**) [30]. Ten flavonoid glycosides were obtained from *R. muricatus*. These included apigenin-8-C-*α*-l-arabinopyranosyl-6-C-*β*-d-glucoside (**200**), apigenin-6-C-*β*-d-glucoside-8-C-*β*-d-glucoside (**201**), kaempferol-3-*O*-(2‴-p-coumarylsophoroside)-7-*O*-*β*-d-glucoside (**202**), kaempferol-3-*O*-(2‴-*E*-caffeoyl sophoroside)-7-*O*-*β*-d-glucoside (**203**), kaempferol-3-*O*-sophoroside-7-*O*-*β*-d-glucoside (**204**), kaempferol-3,7-di-*O*-*β*-d-glucopyranoside (**205**), quercetin-3-*O*-(2‴-*E*-caffeoyl)-*α*-l-arabinopyranosyl-(1→2)-*β*-d-glucoside-7-*O*-*β*-d-glucoside (**206**), quercetin-7-*O*-*β*-d-glucoside (**173**), quercetin-3-*O*-(2‴-*E*-caffeoylsophoroside)-7-*O*-*β*-d-glucoside (207), and quercetin-3-*O*-(2‴-*E*-ferulylsophoroside)-7-*O*-*β*-d-glucoside (**208**) [167]. Additionally, Sadia et al. (2013) identified Tricin7-*O*-*β*-d-lucopyranoside (**209**) in *R. muricatus* [146]. In another investigation, quercetin (**174**), isovitexin (**210**), and isoorientin (**211**) were also detected in *R. arvensis* [62]. Several flavonoids have been isolated from different species of *Anchusa*. In *A. azurea*, four flavonol glycosides, namely astragalin (**212**), isoquercitrin (**213**), rutin (**176**), kaempferol 3-*O*-*α*-rhamnopyranosyl (l‴→6″)-beta-glucopyranoside (**214**), and quercilicosid A (**215**), were isolated [163], and these same compounds were also identified by B. Hu et al. (2020) in their phytochemical analysis of the *A. azurea* extract [152]. Additionally, catechin (**198**) and astragalin (**212**) are the most prevalent components of *A. azurea* [162]. Three flavonoids were isolated from *A. italic* including 5-hydroxy-3′,4′,6,7-tetramethoxyflavone (216), isorhamnetin-3-*O*-*α*-l-rhamnosyl(1–6)-*β*-d-glucopyranoside (**195**), and rutin (**176**) [176]. Two flavonoids, genistein (**217**) and silybin (**218**), were isolated from the ethyl acetate fraction of *A. strigosa*’s [165]. Rutin (**176**) was isolated from *A. undulata* subsp. hybrida [36]. In *E. maritimum*, quercetol (**219**) and kaempferol (**177**) were identified as aglycons and quantified by Conea et al. (2016), while kaempferol **(177**) was found to be the major flavonoid glycoside. Isoquercitrin (**213**) and quercitrin (**175**) were also identified in this study [171]. Similarly, Mejri et al. (2017) reported that kaempferol (**177**) and luteolin (**170**) were the most abundant flavonoids extracted from *E. maritimum* [169], while Pereira et al. (2019) identified naringenin (**220**) as one of the primary constituents in this plant [177]. In another study, Kikowska et al. (2022) isolated three flavonoids, namely kaempferol (**177**), quercitrin (**175**), and rutoside, also known as rutin, quercetin 3 rutinoside (176), from *E. maritimum*, with rutoside (**176**) being identified as the main flavonoid in this plant [170]. Further study is required to fully understand the therapeutic potential of these flavonoids given their wide spectrum of health advantages. Figure 6 illustrates the chemical structure of flavonoids identified in the selected genera.

#### 3.2.5. Alkaloids

Alkaloids are a broad class of chemical molecules that are derived from amino acids and contain nitrogen atoms [178]. Alkaloids have a variety of pharmacological functions such as antiproliferative, antimicrobial, antioxidant, inflammatory, anti-HIV activity, and acetylcholinesterase inhibitor properties that can be exploited in medication development [179]. Reviewing the literature revealed that, among the many known families of alkaloids, pyrrolizidine alkaloids (PAs) were the most abundant in *Anchusa* and *Senesio* species. Although some experimental models have shown that PAs can be toxic, its biological properties are still of great interest and have potential applications in drug discovery programs [180].

**Figure 7:**
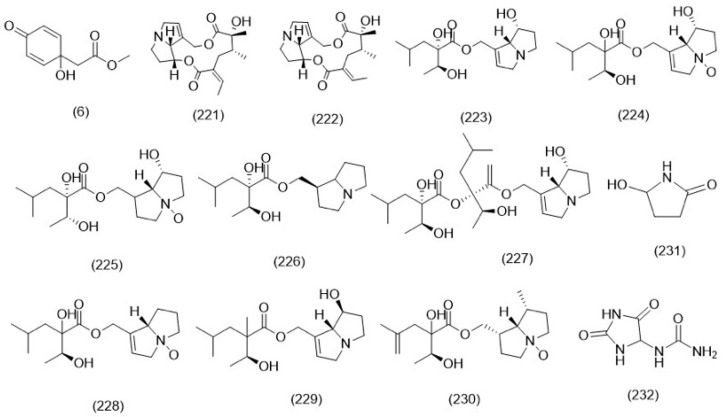
The chemical structure of the alkaloid constituents present in the selected genera.

In a previous study, three alkaloids, jacaranone (**6**), senecionine (**221**), and integerrimine (222), were identified in the extract of *S. leucanthemifolius* [84]. A total of six pyrrolizidine alkaloids were identified in *A. strigosa* including retronecine 2*S*-hydroxy-2*S*(1*S*-hydroxyethyl)-4-methyl-pentanoyl ester (223) and its N-oxide (224), retronecine N-oxide 2*S*-hydroxy-2*S*(1*R*-hydroxyethyl)-4-methyl-pentanoyl ester (225), trachelanthamidine 2*S*-hydroxy-2*S*(1*S*-hydroxyethyl)-4-methyl-pentanoyl ester (226), retronecine 2*S* hydroxy-2*S*(1*S*-hydroxyethyl)-[1′*S*-hydroxyethyl)-4-methylpentanoyl]-4-methyl-pentanoyl ester (227), and supinidine N-oxide 2*S*-hydroxy-2*S*(1*S*-hydroxyethyl)-4-methyl-pentanoyl ester (228) [181]. Heliotridine 2*S*-hydroxy-2*S*-(1*S*-hydroxyethyl)-4-methyl-pentanoyl ester (**229**) and platynecine N-oxide 2*S*-hydroxy-2*S*-(1*S*-hydroxyethyl)-4-methyl-pentanoyl ester (**230**) were two pyrrolizidine alkaloids isolated from *A. strigosa* [130]. Two other alkaloids were also isolated from *A. italica*: 5-hydroxypyrrolidin-2-one (**231**) and allantoin (**232**) [164]. Figure 7 illustrates the chemical structure of alkaloids identified in the selected genera.

#### 3.2.6. Miscellaneous

**Figure 8:**
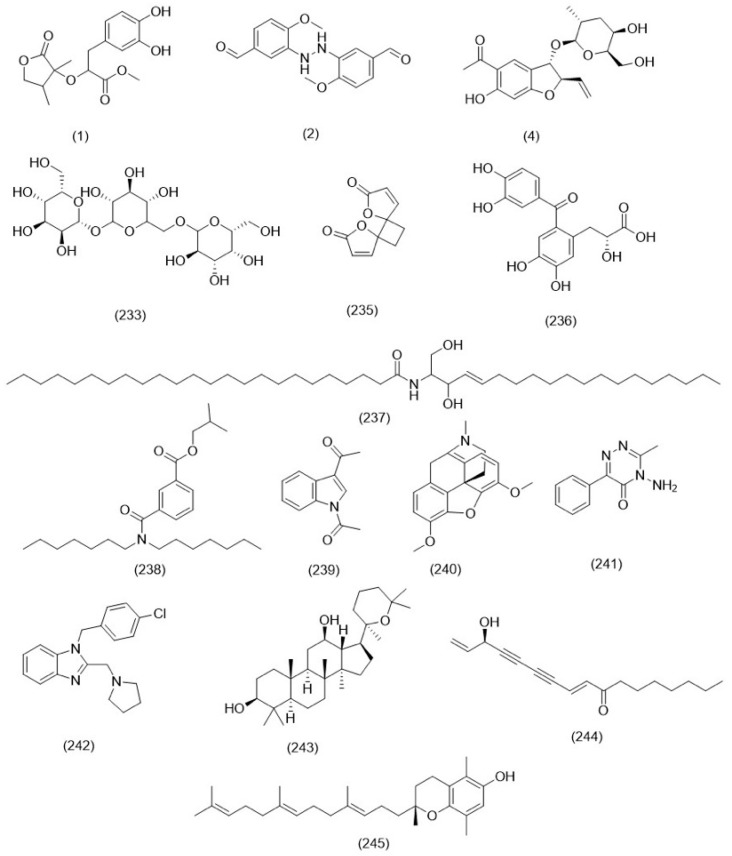
The chemical structure of miscellaneous constituents present in the selected genera.

A recently discovered compound called 2,3-dihydro-3-hydroxyeuparin 3-*O*-glucopyranoside (**4**) has been isolated from *S. glaucus*. This compound belongs to the benzofuran glucoside class [55]. A trisaccharide d-galactopyranosyl-(1→6)-*α*-d-glucopyranosyl-(1 ↔ 1)-*β*-d-glucopyranoside (**233**) was isolated from *C. flammula* [30]. The polysaccharide, poly[3-(3,4-dihydroxyphenyl) glyceric acid (**234**), was also isolated from *A. italica* [182]. Other compounds isolated from *R. muricatus *include a furanone named anemonin (235) [63], a benzophenone named ranunculone C (**236**) [167], 1,3-dihydroxy-2-tetracosanoylamino-4-(*E*)-nonadecene (**237**) [183], and an aromatic lactone named muriolide (**1**) [49]. *E. criticum* was found to contain isobutyl 3-(diheptylcarbamoyl) benzoate (**238**), 1,3-diacetylindole (**239**), thebaine (paramorphine) (**240**), and heterocyclic compounds including metamitron (**241**) and clemizole (**242**) [184]. Two saponins named panaxadiol (**243**) and (*E*)-15-hydroxy 9,16-heptadecadiene-11,13-diyn-8-one (**244**) were isolated from *E. criticum* [149]. *E. maritimum* seed oil is high in tocols, with *β*-tocotrienol (**245**) being the main one [155]. Figure 8 illustrates the chemical structure of the miscellaneous constituents identified in the selected genera.

## 4. Conclusions

This review presents a summary of plant species from eight genera from Jordan, highlighting their chemical constituents, pharmacological properties, and therapeutic relevance. Although many plants are being thoroughly examined for their phytochemical and biological properties, there are still some species that have not been thoroughly investigated. While 245 chemical components have been identified, only a small portion of them have been confirmed to have biological activity such as antioxidant, antimicrobial, cytotoxic, and anti-inflammatory properties, and the mechanisms and pathways of action of these compounds are not fully understood due to most studies being conducted in vitro. Moreover, it is important to conduct further research on the toxicity of plant extracts and isolated compounds as well as their pharmacokinetics and potential drug interactions in vivo. Therefore, additional research is necessary to validate the biological activity of these components and to gain a better understanding of their mechanisms of action, which can provide valuable insights into the potential therapeutic applications of plant-derived compounds.

## Figures and Tables

**Table 1:** Latin names of the selected genera and their species present in Jordan with their common names.

Genus	Species Present in Jordan	Common Name	Reference
*Chrysanthemum* L.	*Ch. segetum* L.	Common chrysanthemum	[6]
	*Ch. coronarium* L.	Corn marigold	
*Onopordum* Vaill. ex L.	*O. alexandrinum* Boiss.	Cotton thistle and artichoke Cotton thistle	[8]
	*O. carduiforme* Boiss.		
	*O. cynarocephalum* Boiss & Blanche.		
	*O. heretacanthum* C.A.Mey.		
	*O. palaestinum* Eig.		
*Phagnalon* Cass.	*Ph. rupestre* L.	African Fleabane	[10]
*Senecio* L.	*S. vulgaris* L. *S. glaucus* L. subsp. *coronopofolius* C. Alexander. *S. flavus* sch.Bip. *S. leucanthemifolius* subsp*. vernalis* Poir.	Common groundsel Decaisne groundsel Bucks horn groundsel	[10]
*Clematis* L.	* C. cirrhosa * L.	Evergreen Virgin’s Bower	[14]
	* C. flammula * L.	Fragrant Bower	
*Ranunculus* L.	* R. arvensis * L.	Corn buttercup	[14]
	* R. asiaticus * L.	Turban buttercup	
	* R. cornutus * DC.	Evil memedotu	
	* R. chius * DC.	Buttercup	
	* R. sceleratus * L.	Cerely-leaved crowfoot	
	* R. muricatus * L.	Spiny-fruited buttercup	
	* R. paludusus * Poir.	Fine-leaved crowfoot	
*Anchusa* L.	*A. undulate* L.,	Common alkanet	[10]
	*A. strigosa* Banks & Sol,	Prickly alkanet	
	*A. azurea* Mill.,	Italian bugloss	
	*A. milleri* Lam.ex Spreng,	Miller’s alkanet	
	*A. aegyptiaca* (L.) A.DC.	Egyptian alkanet	
*Eryngium* L.	*E. creticum* Lam.,	Field Eryngo	[10,14]
	*E. glomeratum* Lam.,		
	*E. falcatum* F. Delaroche	Eryngo	
	*E. maritimum*		

**Table 2:** The antioxidant activities of the selected genera.

Plant	Extracts/Plant Parts Used	Method Used	Results	Reference
*Ch.* *segetum*	Ethanol extract of the flower chloroform, ethyl acetate and *n*-butanol fractions	DPPH CUPRAC	The EtOAc extract demonstrated the highest antioxidant capacity in all assays, with IC_50_ values of 23.58 µg/mL for DPPH activity and 14.85 µg/mL for CUPRAC capacity A050.	[26]
*O. alexandrinum*	Lipid and essential oil from the seed and aerial parts	DPPH	The unsaponifiable fractions of the plant’s seed and aerial parts, volatile oil, exhibited strong antioxidant activity, with a radical scavenging effect of 79.18%, 82.83%, and 81.65%.	[27]
*O. alexandrinum*	Methanol extract of the flowers *n*-hexane, chloroform, ethyl acetate and *n*-butanol fractions	DPPH	The ethanolic extract’s IC_50_ value for its ability to scavenge free radicals was 200 μg/mL. EtOAc fraction displayed the highest activity (IC_50_ of 65 μg/mL), followed by the *n*-butanol fraction (IC_50_ of 150 μg/mL). The *n*-hexane and chloroform fractions had negligible activity.	[28]
* C. cirrhosa *	Essential oil from the aerial parts	TAC DPPH. ABTS^•+^ FRAP CUPRAC	The plant displayed strong antioxidant activity in TAC, FRAP, and ABTS tests, with values of 291.36 mg AAE/g, 119.71 mg TE/g, and 128.91 µg TE/mg, respectively. Moderate antioxidant activity in CUPRAC and DPPH tests, with an IC_50_ value of 5.10 mg/mL.	[29]
* C. flammula *	(100%) methanol and (70%) methanol extracts of the leaves	DPPH FRAP TAC	The total antioxidant capacity of C. flammula leaf methanol/water extract is 642 mg *α*-tocopherol/g extract.	[30]
* C. cirrhosa *	(100%) methanol and (70%) methanol extracts of aerial parts	DPPH. ABTS^•+^ FRAP CUPRAC	The methanol extract had higher TAC activity (138.64 mg AAE/g) than the hydromethanol extract (75.00 mg AAE/g). The difference in ferric ion reducing antioxidant power was slight, with values of 212.42 mg TE/g and 205.15 mg TE/g for methanol and hydromethanol extracts, respectively. Both *C. cirrhosa* extracts showed significant cupric reducing capacity, the hydromethanol extract had slightly higher ABTS^•+^ scavenging capacity (237.80 0.24 µg/mg TE) than the methanol extract.	[31]
* R. sceleratus *	Chloroform, ethyl acetate, *n*-butanol and aqueous fractions	TEAC FRAP DPPH	The soluble fraction of ethyl acetate inhibited the DPPH radical by 80.9%. Total antioxidant activity (1.04) and FRAP value (238.5TE μM/mL).	[32]
* R. sceleratus *	Hydroalcohol and glycerol-ethanol extracts of aerial part	DPPH, TEAC FRAP CUPRAC SNP	IC_50_ of hydro alcohol by different methods: 872.1 μL, 186.7 μL, 103, 61, 297 μM ET/100 mL extract. IC_50_ of glycerol-ethanol by different methods: 988.4 μL, 250.7 μL, 60, 49, 161, 297 μM ET/100 mL extract.	[33]
* R. sceleratus *	Ethanol, chloroform, methanol extract of the root	DPPH ABTS H_2_O_2_	The ethanol extract of *R. sceleratus* exhibited the highest H_2_O_2_ scavenging activity and displayed optimal ABTS and DPPH radical scavenging activity.	[34]
* R. sceleratus *	Methanol extract of Shoot and roots	DPPH	The antioxidant activity of the *R. sceleratus* has an IC_50_ value of 0.37 mg/mL and 0.34 mg/mL for the shoot and root, respectively	[35]
*A. undulata*	Methanol extract of aerial parts	DPPH ABTS FRAP CUPRAC *β*-carotene-linoleic acid method Phosphomolybdenum method Hydroxyl radical scavenging activity Superoxide anion scavenging activity Nitric oxide radical scavenging activity	The methanol extract exhibited high inhibition values for linoleic acid oxidation and had a total antioxidant capacity of 1.531 mmol AAEs/g extract, DPPH scavenging activity of 2.086 mmolTEs/g extract, ABTS assay of 0.112 mmol TEs/g extract, hydroxyl radical scavenging activity of 0.208 mmol MEs/g extract, and NO scavenging activity of 3.866 mmol TEs/g extract. The extract displayed concentration-dependent chelating activity and reduction of Cu(II) ability, with 0.081 mmol TEs/g extract. The reducing power assays showed values of 0.329 mmol TEs/g extract for potassium ferric cyanide and 0.425 mmol TEs/g extract for FRAP.	[36]
*A*. undulata L. subsp. hybrida**	Methanol extract of roots and aerial parts	ABTS DPPH	IC_50_ values for DPPH were 239.47 and 292.04 µg/mL and for ABTS were calculated as 41.15 and 32.3 µg/mL for roots and aerial parts respectively.	[37]
* A. strigosa *	Methanol extract of the flower	DPPH *β*-carotene bleaching assay	IC_50_ value 43.75 µg/mL against DPPH radical. IC_50_ value for β-carotene bleaching 425.8 µg/mL	[38]
* A. italica *	Hydro-ethanol extract of the roots	FRAP DPPH TAC	Root extract displayed strong iron reduction capacity in the FRAP assay (IC_50_ 0.11 µg/mL). IC_50_ values in the DPPH test were 0.11 µg/mL for root extract and 0.14 µg/mL for leaf extract, lower than those for ascorbic acid (IC_50_ 0.16 µg/mL) and BHT (IC_50_ 0.20 µg/mL). TAC values were 0.51 and 0.98 mg AAE/g extract for the leaf and root extracts, respectively.	[39]
* E. creticum *	Aqueous extract of leaves and stems	ABTS H_2_O_2_	*E. creticum* leaves and stems (100 g fresh) provide antioxidants equivalent to 78.50 mg and 50.42 mg of vitamin C. Inhibition of H_2_O_2_ by 25 mg/mL of *E. creticum* leaves was 96%.	[40]
* E. creticum *	Ethanol and aqueous extracts from both leaves and stems	DPPH Ferrozine H_2_O_2_	The EtOH extract of *E. creticum* leaves and stems exhibited higher antioxidant activity. The IC_50_ values for the EtOH extract were 0.18 mg for leaves and 3 mg for stems. The EtOH extract also showed better chelating activity than the aqueous extract, with IC_50_ values of 0.4 mg for EtOH leaves and 0.5 mg for aqueous leaves. The IC_50_ values for H_2_O_2_ were 2.4 mg for leaves and 12 mg for stems.	[41]
* E. creticum *	Ethanol (40%, 80% and 100%) extract of the plant	DPPH Chelating effects on ferrous ions	40% ethanol extract possessed the highest iron chelating activity (87.92%) while the 80% ethanol extract showed 89.92% DPPH scavenging activity.	[42]
* E . maritimum *	Essential oils from aerial parts	DPPH ABTS	The total essential oil exhibited strong antioxidant activity with IC_50_ values of 5.9 mg/mL and 0.7 mg/mL for DPPH and ABTS radical-scavenging abilities, respectively. The oxygenated fraction demonstrated the best radical-scavenging effect, with IC_50_ values of 8.8 mg/mL for DPPH and 1.34 mg/mL for ABTS radical.	[43]
* E . maritimum *	Volatile fraction of the fruits	DPPH ABTS	The volatile extracts displayed significantly higher antioxidant activity than Trolox, with IC_50_ values for DPPH and ABTS radical-scavenging capacity at least twice lower than those observed for the reference compound.	[44]
*E. maritimum*	Water, methanol acetone, and ethyl acetate extract of the Aerial parts	DPPH ABTS	Different extracts were active using DPPH, the IC_50_ value ranged from 1.247 to 31.19 mg/mL of solution. ABTS values ranged from 0.109 to 3.36 mg AA/g.	[45]
* E. maritimum *	Conventional reflux extraction (water and ethanol) and alternative techniques for aerial parts	DPPH assay Xanthine oxidase assay	Aqueous extracts prepared by reflux, microwave-assisted, and ultrasound-assisted exhibited the highest antioxidant activity in the DPPH assay (>70%). Reflux 80% ethanol and supercritical fluid extraction showed the best response to the xanthine oxidase assay (105% and 137%).	[46]
* R. * * muricatus *	Ethyl acetate soluble fraction of methanol extract of the whole plants	DPPH Lipoxygenase inhibition assay Urease inhibition assay	Muricazine exhibited greater effectiveness in scavenging DPPH radical, with an IC_50_ value of 42.1 μM compared to the positive control. It displayed moderate inhibitory potential against lipoxygenase (65.2 μM) and urease (54.8 μM).	[47]

**Table 3:** The antimicrobial activities of the selected genera.

Plant	Extract/Part Used	Method Used	Results	Reference
* Ch. coronarium *	Essential oils from flower head	Agar diffusion plate assay	The growth of *Alternaria* sp., *A. flavus*, and *P. ultimum* was significantly reduced by more than 80%.	[52]
* Ch. coronarium *	Essential oils from flower head	Paper-disk diffusion method	Good antibacterial properties against *B. subtilis* and *S. aureus* with zone of inhibition (mm) values: 19 and 20.	[53]
*S. glaucus*	Ethyl acetate soluble fraction of methanol extract of the aerial parts	Agar diffusion plate assay	2,3-dihydro-3bhydroxyeuparin 3-*O*-glucopyranoside exhibited strong antibacterial properties (MIC = 8 μM) against *E. Coli*, *B. subtilis*, and *S. aureus*. Antifungal activity against *C. albicans* and *C. tropicalis* (MIC = 8 μM).	[54]
*S. vulgaris*	Aerial parts methanol extract *n*-hexane, dichloromethane, ethyl acetate, and *n*-butanol fractions	Microdilution technique	MICs of 0.5 mg/mL of methanol extract for *B. subtilis* and 0.125 mg/mL for *S. aureus.* The methanol extracts exhibited limited efficacy against dermatophytes, displaying MIC values of 0.5 mg/mL. The n-hexane fractions displayed effectiveness against *T. tonsurans*, with an inhibitory concentration of 0.031 mg/mL.	[55]
*S.* *leucanthemifolius*	Aerial parts methanol extract *n*-hexane, dichloromethane, ethyl acetate, and *n*-butanol fractions	Microdilution technique	The ethyl acetate extract showed the most potent efficacy against *S. aureus*, with an MIC value of 31.25 mg/mL, and against *C. albicans*, with an MIC value of 125 mg/mL. *n*-Hexane extract showed noteworthy efficacy in combating the dermatophytes *T. tonsurans* and *M. gypseum* with an MIC value of 125 mg/mL.	[56]
*C. flammula*	Ethanol extract of leaves	Agar diffusion plate assay	Six bacterial species were inhibited: *E. faecalis*, *P. mirabilis*, *L. monocytogenes*, *P. aeruginosa*, *C. jejuni*, *C. xerosis*, growth inhibition zone is given in mm: 10.6, 9.3, 13.5, 10.4, 10.3, respectively.	[57]
*C. flammula*	Ethanol extract of leaves	Broth microdilution method In vitro biofilm inhibition assay Cell surface hydrophobicity assay Germ tube elongation assay	MIC_50_ against *C. albicans* strains 164.89 µg/mL. A dose-dependent reduction in cell surface hydrophobicity was observed. Reduction in both germ tube and hyphae.	[58]
* R. sceleratus *	Essential oils	Agar well diffusion method	The extract displayed modest antibacterial efficacy against *P. aeruginosa*, *E. faecalis*, and *S. aureus*; antifungal activity against *C. albicans*, with MIC values of 8, 8, 8, and 34 µg/mL, respectively	[59]
*R. sceleratus*	Methanol, aqueous and chloroform extracts of the leaves	Agar well diffusion method Broth macro dilution method	Chloroform extract exhibited the maximum activity with a halo of 23 mm diameter inhibition against *T. mentagrophytes* followed by *T. rubrum* (22 mm), *M. fulvum* (21 mm), *M. gypseum* (18 mm), and *T. tonsurans* (15 mm). The methanol extracts produced inhibition zones of 21, 16, 17, 17 and 10 mm, respectively for *T. mentagrophytes*, *M. gypseum*, *T. rubrum*, *M. fulvum*, and *T. tonsurans*.	[60]
* R. sceleratus *	Aqueous extracts of the leaves	% of inhibition in colony diameter	Mycelial inhibition (%) for *A. brassicae* and *A. brassicicola* were 93.42 and 85.16, respectively.	[61]
* R. sceleratus *	Ethanol and methanol extracts of roots	Disc diffusion assay	MIC of the ethanol extract ranged from 21 to 17.67 mg/mL; methanol extract ranged from 13.67 to 14 mg/mL against *A. baumannii*, *A. niger*, *B. subtilis*, *P. aeruginosa*, *S. aureus*, and *S. cerevisiae.*	[62]
* R. * *arvensis*	Aqueous extract of aerial parts	Diffusion discs methods	Strong antifungal action against *C. albicans*. The growth inhibition zone measured 21 mm.	[63]
* R. arvensis *	Essential oils	Paper disk diffusion technique	Significant activity against *Escherichia coli*, *S. aureus*, *Enterobacter* sp., and *P. vulgaris*, maximal inhibition zones ranging from 15 to 21 mm.	[64]
* R. * *arvensis*	Whole plants methanol extract dichloromethane, ethyl acetate and *n*-butanol fractions	Agar tube dilution protocol Microplate Alamar Blue assay	The dichloromethane fraction exhibited antibacterial effects against *B. subtilis*, *S. aureus*, *P. aeruginosa*, and *S. typhi*; fungicidal effects against *M. canis* and *F. solani*.	[65]
* R. muricatus *	Fractions from *n*-hexane, chloroform, ethyl acetate and ethanol extract	Agar well diffusion method Agar dilution method	Ethyl acetate fraction exhibited the most potent antibacterial efficacy against *S. aureus* with an MIC of 0.119 µg/mL. *n*-Hexane fraction showed the strongest antifungal efficacy against *A. niger*.	[66]
* A. strigosa *	*n*-Hexane extract of flowers	Disc diffusion method	The total lipids showed a notable antibacterial effect at different doses (0.01–10 mg/mL), with greater activity observed in Gram-positive bacteria in the order of *P. aeruginosa*, *S. faecalis*, *S. aureus*, and *B. subtilis*. The effect on Gram-negative bacteria followed the sequence *Proteus* sp., *E. coli*, *Enterobacter* sp., and *Klebsiella* sp.	[67]
* A. strigosa *	Essential oil extracted from the flowers, Fixed oil extracted from the flowers	Disc diffusion method	The essential oil had antibacterial efficacy against both Gram-positive and Gram-negative bacteria including *P. aeruginosa*, *Proteus* sp., and *S. faecalis*. Good efficacy was shown by fixed oil against *P. aeruginosa*, *Proteus* sp., and *Klebsiella* sp., particularly at higher concentrations of 500 μg/mL.	[68]
* A. strigosa *	Aqueous and ethanol extract	Agar well diffusion method	The alcohol extract of *A. strigosa* exhibited a pronounced inhibitory effect on resistant bacteria, *S. salivarius* and *S. pyogenes*, with inhibition zone diameters of 27.0 and 26.0 mm, respectively, compared to its aqueous extract.	[69]
* A. azurea *	Aerial parts methanol extract *n*-hexane, chloroform, ethyl acetate fraction	Beta-latamase inhibition assays	At a concentration of 10 mg/mL, both the crude extract and ethyl acetate extract of *A. azurea* showed a very high percentage of inhibition, ranging from 58% to 68%.	[70]
* A. azurea *	Ethanol extract of leaves and roots	Agar disk diffusion microdilution assay.	Both the leaves and roots extracts demonstrated inhibitory effects against four strains of *E. coli*, two strains of *K. pneumoniae*, and *coagulase-negative Staphylococcus*, with zone of inhibition diameters ranging from 11.00 to 16.00 mm for the root extract and 11.67 to 14.33 mm for the leaf extract.	[39]
* E. creticum *	Aqueous and ethanol extract of leaves and stem	Broth microdilution assay	The aqueous and ethanol extracts of the leaves were effective against *S. epidermidis*, with MIC values of 5 mg/mL and an MBC of 10 mg/mL for the ethanol extract. *E. faecalis* was sensitive to the extracts but showed greater resistance in the second test phase. *S. aureus* exhibited consistent sensitivity, while *E. coli* was highly resistant. *P. aeruginosa* displayed alternate resistance and had higher values in the second period extracts. The MBC and MIC for the leaves were both 244 mg/mL.	[71]
*E. creticum*	Petroleum ether and methanol extracts of leaves	Mycelial growth inhibition Spore germination tests	In the mycelia growth inhibition test, the petroleum ether extracts exhibited antimycotic activity ranging from 33% to 98%, while the methanol extracts ranged from 3% to 75%. Petroleum ether showed high activity against only *B. cinerea* and *F. oxysporum*, with more than 95% inhibition of spore germination.	[72]
*E. glomeratum*	Essential oil extracted from aerial parts	Agar dilution method	MIC values of up to 2 μg/mL.	[73]
* E . maritimum *	Essential oils extracted from fruits and leaves	Broth microdilution assay	The essential oils from both the leaves and fruits were effective against *T. mentagrophytes*, with MIC values of 1.56 mg/mL and 7.5 mg/mL, respectively as well as *S. aureus*, with MIC values of 12.5 mg/mL and 60 mg/mL, respectively. The basal leaf essential oil exhibited moderate antibacterial activity against *C. albicans* and *E. coli*, with MIC values of 12.5 mg/mL and 25 mg/mL, respectively.	[74]
* E . maritimum *	Roots methanol extract Acetone, ethyl acetate and *n*-butanol fractions	Disc diffusion method	Inhibition was shown by all extracts against *B. cereus*, *S. aureus*, *E. coli*, and *L. monocytogenes.* Methanol and *n*-butanol extracts were effective against *P. aeruginosa*. The ethyl acetate extract exhibited the maximum activity against all test fungi, with *A. flavus* showing the greatest inhibition zone of 10 mm at a concentration of 50 mg/mL.	[75]
*E. maritimum*	Ethanol extracts from leaves and roots	Method of series dilutions	The antifungal activity of *E. Maritimum* was most prominent against *T. mentagrophytes* dermatophyte strains, with MIC values ranging from 40 to 100 mg/mL.	[76]
* E. maritimum *	Conventional reflux extraction (water and ethanol) and alternative techniques for aerial parts	Agar dilution method	The extracts demonstrated antibacterial activity against five species including *P. acnes*, *Streptococcus bovis*, *S. pyogenes*, *S. dysgalactiae*, and *S. pneumoniae*. The supercritical fluid extraction was effective in inhibiting all strains of *P. acnes* at 400 mg/L, while the reflux ethanol extract was able to inhibit the clinical strain N896 of *P. acnes*.	[46]
*E. falcatum*	Aqueous, methanol, and ethyl acetate extracts of the root and aerial parts Essential oils	Microbroth dilution technique	*E. Maritimum* essential oil had moderate antimicrobial potential, with *K. pneumoniae* and *P. mirabilis* being sensitive, *S. pyogenes* was resistant. Acetone extract was the most efficient, followed by the EtOAc and MeOH extracts, while the H_2_O extract had no activity. *E. falcatum* aerial and root parts had moderate antibacterial activity against Gram-positive strains.	[77]

**Table 4:** The cytotoxic and antiproliferative activities of the selected genera.

Plant	Extract/Part Used	Method Used	Results	Reference
*Ch. coronarium*	Essential oils of flower head	MTT cell proliferation assay	The essential oil inhibited the growth of various tumor cell lines (Caco-2, T47D, MCF-7, HeLa), with LD_50_ values ranging from 43 to 110 µg/mL.	[53]
*Ch. coronarium*	The methanol extract of aerial parts	MTT cell proliferation assay	The IC_50_ values of the *Ch. coronarium* extract against WM1361A, CACO-2, HRT18, MCF-7, T47D, and A375.S2 ranged from 75.8 to 138.5 μg/mL.	[80]
*O. cynarocephalum*	Water extract of aerial parts	MTT cell proliferation assay Flow cytometry analysis TUNEL assay Western blot analysis	The extract dose-dependently inhibited HCT-116 cell growth (IC_50_ 0.18 mg/mL) more effectively than HT-29 cells (IC_50_ 1.8 mg/mL). It upregulated p53 and Bax while suppressing Bcl-2, inducing apoptosis.	[81]
*O. cynarocephalum*	Acetone and chloroform extracts of the aerial parts	MTT cell proliferation assay DNA analysis by COMET assay Caspase colorimetric assay Western blot analysis	Acetone and chloroform extracts inhibited the M14, A2058, and A375 cell lines, with higher potency against A375. IC_50_ values on A375 cells were 21.32 µg/mL (acetone) and 10.12 µg/mL (chloroform). Both extracts increased caspase-3 activity, inducing apoptosis via PTEN inhibition and Hsp70 downregulation.	[82]
*S. vulgaris*	Aerial parts methanol extract (*n*-hexane, dichloromethane and ethyl acetate) fractions	Sulforodamine B assay	Caco-2 exhibited the highest sensitivity to the methanolic and CH_2_Cl_2_ extracts of *S. vulgaris*, with IC_50_ values of 34 mg/mL and 5 mg/mL, respectively.	[83]
*S. leucanthemifolius*	Aerial parts methanol extract (*n*-hexane, dichloromethane, and ethyl acetate, *n*-butanol) fractions	Sulforhodamine B assay	The large cell carcinoma (IC_50_ 20.1 μg/mL) and colorectal adenocarcinoma (IC_50_ 36.37 μg/mL) were inhibited by dichloromethane extracts, the *n*-hexane extract demonstrated notable activity against hepatocellular carcinoma (IC_50_ value 30.88 μg/mL).	[84]
*C. flammula*	Ethanol extract of leaves	MTT cell proliferation assay	Cytotoxic potential on CHL and PLC with IC_50_ values of 58.5 and 47.3 µg/mL, respectively).	[85]
* E. creticum *	Aqueous, methanol, and ethyl acetate from fresh leaves and stems	XTT cell viability assay	The methanol extracts of the two plant parts inhibited MCF7 cell growth by 72% and 68% after 48 h of treatment. The aqueous and ethyl acetate extracts (at 2.5 mg/mL) of the leaves and stems showed no cytotoxicity.	[86]
* E. creticum *	Ethanol extracts of leaves, stems, roots, and whole plant	Neutral red assay DNA fragmentation assay	All plant parts inhibited HeLa cell viability (0–250 M). The ethanol extract of the second harvest leaves displayed the strongest potency with an IC_50_ value of 47.24 μg/mL at 48 h.	[87]
*E. glomeratum*	Petroleum ether extracts of the aerial parts	MTT cell proliferation assay	Cytotoxicity of *E. glomeratum* against cancer macrophage-like cell lines (J774) was 1.11 μg/mL, with selectivity indices of 2.1.	[88]
*E. maritimum*	Aqueous extract of aerial and root parts	MTT cell proliferation assay	*E. maritimum* showed IC_50_ values of 32.42 µg/mL and 35.01 µg/mL for the aerial and root parts, respectively, in HepG2 cells. In contrast, the IC_50_ values were 50.00 µg/mL and 30.25 µg/mL for the aerial and root parts, respectively, in Hep2 cells. The LDH method yielded the lowest IC_50_ values for Hep2 cells, which were 51.67 µg/mL and 34.32 µg/mL for the aerial and root parts, respectively.	[89]
*Ch. coronarium*	Ethyl acetate fraction of methanol extracts of aerial parts	XTT cell viability assay	Campesterol exhibited a concentration-dependent inhibition of bFGF-induced proliferation and tube formation in HUVECs. Campesterol was effective in disrupting bFGF-induced neovascularization in chick chorioallantoic membrane (CAM) in vivo.	[90]
*S. glaucus*	Ethyl acetate fraction of the aerial parts	MTT cell proliferation assay	Cytotoxic activity against PANC-1 cancer cell lines was (IC_50_ 7.5 μM).	[54]

**Table 5:** The anti-inflammatory activities of the selected genera.

Plant	Extract/Part Used	Method Used	Results	Reference
*Ch. coronarium* and *Ch. segetum*	Essential oil of flowerheads	Phosrithong and Nuchtavorn method	IC_50_ values of *Ch. coronarium* and *Ch. segetum* against the 5-lipoxygenase enzyme were 0.151 and 0.017 mg/mL, respectively.	[94]
*O. cynarocephalum*	Water extracts of the aerial part	In vitro model of ET-induced inflammation in SCp2 cells. In vivo model of ET-induced paw edema	The extract was found to inhibit ET-induced IL-6, gelatinases, and NF-B activation in SCp2 mammary epithelial cells. Extract demonstrated a significant reduction in paw edema in a model of ET-induced edema.	[95]
*C. flammula*	Ethanol extract of leaves	Assessment of the mucus content in the stomach wall using the indomethacin ulcer model Inhibition of H^+^/K^+^-ATPase Determination of myeloperoxidase activity	The proton pump and MPO activities were significantly inhibited by the ethanol extract, with decreases of 90% and 99%, respectively.	[96]
* R. sceleratus *	Ethanol extract of aerial parts and roots	Nitrite concentration in LPS-stimulated RAW 264.7 macrophage cell line	An inhibitory effect was seen in relation to concentration in all extracts; the aerial parts extract showed the most potent inhibition (IC_50_ = 22.08 ± 1.32 g/mL), even surpassing indomethacin.	[97]
* R. muricatus *	Aqueous and methanol extract of whole plant	Carrageenan and egg albumin induced paw edema in mice Acetic acid induced writhing Formalin induced paw licking in mice models	At 150 mg/kg, the maximum dosage, the extract reduced paw edema caused by carrageenan and egg albumin. The extract at the same dosage significantly reduced formalin-induced paw licking and acetic acid-induced abdominal constrictions and hind limb stretching.	[98]
* A. strigosa *	Aqueous and methanol extracts of whole plant	Complete Freund’s adjuvant (CFA)-induced	Both methanol and aqueous extracts were found to significantly reduce paw edema, arthritis index, and body weight loss. The extracts decreased the elevated levels of serum WBC in rats induced with CFA.	[99]
* A. azurea *	Methanol extracts of aerial parts and the roots fractionated with *n*-hexane, *n*-butanol	Carrageenan-induced paw edema in rats	The methanol extract of aerial parts displayed 30% anti-inflammatory activity at a 200 mg/kg bw dose. *n*-Butanol fraction of the methanol extract exhibited the highest anti-inflammatory activity with a 42% reduction at the same dosage.	[100]
*E. maritimum*	Ethanol extract of aerial parts	Turpentine oil-induced acute inflammation model in rats	The extract exhibited anti-inflammatory effects by decreasing the proliferation and activity of total leukocytes and neutrophils. The extract significantly reduced the synthesis of NO.	[101]
*E. maritimum*	Methanol extract of leaves	Cyclooxygenase-1 assay	Anti-acetylcholinesterase activity was 65.34%.	[102]
*E. maritimum*	Ethanol extracts from the aerial parts and roots	*p*-Benzoquinone-induced writhing test Carrageenan-induced paw edema TPA-induced ear edema tests	The ethanol extract of aerial portions significantly reduced ear edema induced by TPA with a 58.8% inhibitory ratio. Inhibitory ratio of aerial parts against *p*-benzoquinone-induced writhings in mice was 55.8%. Aerial parts had a 36.1% inhibitory ratio against carrageenan-induced paw oedema in mice.	[22]
* A. azurea *	Rosmarinic acid isolated from *n*-butanol fraction	Carrageenan-induced paw edema in rats	At a dosage of 50 mg/kg bw, the anti-inflammatory effects of rosmarinic acid were found to be similar to those of indomethacin.	[100]

**Table 6:** The antidiabetic effect of the selected genera.

Plant	Extract/Part Used	Method Used	Results	Reference
*S.* *leucanthemifolius*	Methanol extract of aerial parts fractionated with *n*-hexane, dichloromethane, ethyl acetate *n*-butanol	*α*-Amylase inhibition assay	At 0.05 mg mL^−1^, the dichloromethane extract inhibited *α*-amylase by 56.6%; the *n*-butanol extract inhibited *α*-amylase by 89.2%.	[56]
* C. cirrhosa *	100%methanol and 70%methanol of aerial parts	*α*-Amylase inhibition assay *α-*Glucosidase inhibition assay	The methanol extract had a greater inhibitory effect on *α*-amylase (36.63%) compared to the hydromethanol extract (24.70%). The hydromethanol extract had a significantly higher *α*-glucosidase inhibition rate (40.93%) compared to the methanol extract (14.03%).	[31]
*A. undulata*	Methanol extract of aerial parts	*α*-Amylase inhibition assay *α*-Glucosidase inhibition assay	Compared to α-amylase (0.193 mmol ACEs/g extract), the methanol extract showed stronger α-glucosidase inhibition (0.219 mmol ACEs/g extract).	[36]
*A. undulata * L. subsp. *hybrida*	Methanol extract of roots and aerial parts	*α*-Amylase inhibition assay *α*-Glucosidase inhibition assay	With increasing concentrations, the methanol extract of the roots and aerial parts of herbs inhibited both *α*-glucosidase and *α*-amylase.	[37]
*A. strigosa*	Aqueous extract of flowers	Antidiabetic effect in streptozotocin-induced diabetic rats	The aqueous extract had an antidiabetic effect in diabetic rats, reducing the blood glucose levels and improving the serum insulin levels in a dose-dependent manner. Cholesterol and triglyceride levels were significantly lower, and hepatic glycogen levels increased.	[107]
*E. creticum*	Aqueous extracts of aerial parts	MTT cell proliferation assay of *β*-cell proliferation	Aqueous extracts increased beta-cell proliferation (0.001–1.0 mg mL^−1^) in a dose-dependent manner	[108]

**Table 7:** The antiulcer effect of the selected genera.

Plant	Extract/Part Used	Method Used	Results	Reference
* A. strigosa *	Ethanol extract of the roots partitioned with petroleum ether, chloroform, and *n*-butanol	Ethanol-induced ulcer model	The petroleum ether-soluble fraction, which provided 91% protection, was the most effective at lowering the ulcer index. The *n-butanol-soluble* fraction only provided 65% protection; the chloroform-soluble fraction provided 86% protection; the water-soluble portion failed to adequately shield the stomach from the inflammatory agent.	[113]

**Table 8:** The neuroprotective effect of the selected genera.

Plant	Extract/Part Used	Method Used	Results	Reference
*A. undulata*	Methanol extract of aerial parts	Anticholinesterase activity Anti-butyrylcholinesterase activity	The extract displayed inhibitory effects on both AChE and BChE, with values of 2.238 and 1.239 μmol GALAEs/g extract, respectively.	[36]
*A. undulata L. subsp. hybrida*	Methanol extract of roots and aerial parts	Anticholinesterase activity Anti-butyrylcholinesterase activity	The methanolic extract’s cholinesterase inhibitory effects on AChE and BChE were measured in galantamine equivalents and were found to be 2.238 and 1.239 μmol GALAEs/g extract, respectively.	[37]
*C. cirrhosa*	100% methanol and 70% methanol of aerial parts	Anticholinesterase activity Anti-butyrylcholinesterase activity	*C. cirrhosa* extracts showed significant AChE inhibition, with the hydromethanolic extract demonstrating higher activity levels compared to the methanolic extract. The methanol extract exhibited higher BChE inhibition rates; the hydromethanolic extract had a greater overall inhibitory effect on both enzymes. Both extracts were less effective against BChE compared to the positive control galantamine.	[31]

**Table 9:** Miscellaneous activity of the selected genera.

Plant	Extract/Part Used	Method Used	Results	Reference
* R. muricatus *	Methanol extract	Langendorff perfused heart apparatus	The methanol extract at doses between 1 ng and 10 mg increased the perfusion pressure and force of contraction. The crude extract also significantly increased the heart rate at doses between 1 ng and 1 μg.	[117]
*A. italica*	Ethanol extract of whole plant	Western blot	The *A. italica* extract increased post-MI mice survival rates, with 30 mg/kg and 50 mg/kg doses resulting in 73% and 80% survival rates, respectively. Treatment at these doses reduced the infarct size and TNF-, IL-1, and IL-6 levels. Treatment suppressed the activation of the PI3K/Akt/mTOR signaling pathway.	[118]
*A. italica*	70% ethanol extract of flowers	The ischemia model involved bilateral occlusion of carotid arteries	*A. italica*’s hydroalcohol extract scavenged the free radicals produced by ischemia/reperfusion.	[119]

## Data Availability

Not applicable.

## Abbreviations

12(S)HHTrE	12-Hydroxyheptadecatrienoic acid
5(S)-HETE	5-Hydroxyeicosatetraenoic acid
* A. baumannii *	* Acinetobacter baumannii *
*A. brassicae*	*Alternaria brassicae*
*A. brassicicola*	*Alternaria brassicicola*
*A. flavus*	*Aspergillus flavus*
*A. niger*	*Aspergillus niger*
A375	Human melanoma cell line
A375.S2	Human Melanoma cell line
A549	Adenocarcinomic human alveolar basal epithelial cells
ABTS	2,2′-Azino-bis(3-ethylbenzothiazoline-6-sulfonic acid
AChE	Acetylcholinesterase
ALP	Alkaline phosphatase
ALT	Alanine aminotransferase
AST	Aspartate aminotransferase
*B. cinerea*	* Botrytis cinerea *
*B. subtilis*	*Bacillus subtilis*
*B. cereus*	*Bacillus cereus*
BChE	Butyrylcholinesterase
*C. jejuni*	*Campylobacter jejuni*
*C. xerosis*	*Corynebacterium xerosis*
*C. albicans*	*Candida albicans*
C32	Amelanotic melanoma
CACO-2	Colorectal adenocarcinoma
CAT	Catalase
CCl_4_	Chemokine (C-C motif) ligands 4
CH_2_Cl_2_	Methylene chloride
COR-L23	Large cell cancer
CUPRAC	Cupric ion-reducing antioxidant capacity
DPPH	2,2-Diphenyl-1-picrylhydrazyl
*E. coli*	*Escherichia coli*
*E. faecalis*	*Enterococcus faecalis*
*E. faecalis*	*Enterococcus faecalis*
ECC-1 cells	Endometrial cancer cells
ET-induced inflammation	Endotoxin-induced pro-inflammatory markers
ESI	Electrospray ionization
EtOAc	Ethyl acetate
*F. oxysporum*	* Fusarium oxysporum *
*F. solani*	*Fusarium solani*
FRAP	Ferric reducing ability of plasma
FTICR	Fourier transform ion cyclotron resonance mass spectrometry
FT-IR	Fourier transform infrared spectroscopy
GC-MS	Gas chromatography-mass spectrometry
GPX	Glutathione peroxidase
HCT-116	Human colon cancer cell line
HCT-15	Colorectal adenocarcinoma
HEK293	Human embryonic kidney 293 cells
HeLa	Cervical cancer cells
Hep2	Human laryngeal epidermoid carcinoma
HepG-2	Hepatocellular carcinoma
HPLC	High-performance liquid chromatography
HR-MS	High-resolution mass spectrometry
HRT18	Human rectum adenocarcinoma
HSL	The hormone-sensitive lipase
HT-29	Human colon cancer cell line
IC_50_	The half-maximal inhibitory concentration
iNOS	Inducible nitric oxide synthase
*K. pneumoniae*	*Klebsiella pneumoniae*
*L. monocytogenes*	*Listeria monocytogenes*
LC-MS	Liquid chromatography-mass spectrometry
LD	Lactate dehydrogenase
LPO	lipid peroxidation
LPS	Lipopolysaccharides
LTB4	Leukotriene B4
*M. canis*	*Microsporum canis*
*M. fulvum*	*Microsporum fulvum*
*M. gypseum*	*Microsporum gypseum*
MAE	Microwave-assisted extraction
MCF7	Breast cancer epithelial cell line
MDA-MB-231	Human breast carcinoma
MDBK	Madin–Darby bovine kidney cells
MeOH	Methanol
MI	Myocardial infarction
MIC	Minimum inhibitory concentration
MRC-5	Human fetal lung cell line
MTS	3-(4,5-Dimethylthiazol-2-yl)-5-(3-carboxymethoxyphenyl)-2-(4-sulfophenyl)-2H-tetrazolium
MTT	Colorimetric assay for assessing cell metabolic activity
*n*-BuOH	1 Butanol
NMR	Nuclear magnetic resonance
NO	Nitrogen oxide
ORAC	Oxygen radical absorbance capacity
*P. aeruginosa*	*Pseudomonas aeruginosa*
*P. mirabilis*	*Proteus mirabilis*
*P. ultimum*	*Pythium ultimum*
*P. vulgaris*	*Proteus vulgaris*
*P. acnes*	*Propionibacterium acnes*
PC-3	Human prostate cancer cell line
PMNL	Polymorphonuclear leukocytes
RAW 264.7	Murine macrophage cell line
RKO cancer cells	Colorectal cancer cell line
*S. aureus*	*Staphylococcus aureus*
*S. bovis*	*Streptococcus bovis*
* S. cerevisiae *	* Saccharomyces cerevisiae *
*S. dysgalactiae*	*Streptococcus dysgalactiae*
*S. epidermidis*	*Staphylococcus epidermidis*
*S. pyogenes*	*Streptococcus pyogenes*
*S. typhi*	*Salmonella typhi*
SFE	Supercritical fluid extraction
SNP	Silver nanoparticle assay
SOD	Superoxide dismutase
spp	Species
*St. salivarius*	*Streptococcus salivarius*
*T. mentagrophytes*	*Trichophyton mentagrophytes*
*T. rubrum*	*Trichophyton rubrum*
*T. tonsurans*	*Trichophyton tonsurans*
T47D	Human breast ductal carcinoma
TBA	Thiobarbituric acid assays
TEAC	Trolox equivalent antioxidant capacity assay
UAE	Ultrasound-assisted extraction
UV	Ultraviolet
WEHI	Fibrosarcoma cell line
WM1361A	Primary melanoma cell line
